# Score Matching for Differential Abundance Testing of Compositional High‐Throughput Sequencing Data

**DOI:** 10.1002/sim.70534

**Published:** 2026-04-07

**Authors:** Johannes Ostner, Hongzhe Li, Christian L. Müller

**Affiliations:** ^1^ Computational Health Center Helmholtz Munich Neuherberg Germany; ^2^ Institut für Statistik Ludwig‐Maximilians‐Universität München Munich Germany; ^3^ Department of Biostatistics, Epidemiology and Informatics Perelman School of Medicine, University of Pennsylvania Philadelphia Pennsylvania USA; ^4^ Center for Computational Mathematics Flatiron Institute New York New York USA

**Keywords:** compositional data, differential abundance, generative model, microbiome, score matching, single‐cell RNA sequencing

## Abstract

The class of a‐b power interaction models, proposed by [1], provides a general framework for modeling sparse compositional data with pairwise feature interactions. This class includes many distributions as special cases and enables modeling of zero entries through power transformations, making it particularly suitable for modern high‐throughput sequencing data with excess zeros, including single‐cell RNA‐Seq and microbial amplicon data. Here, we present an extension of this class of models that allows inclusion of covariate information, thus enabling accurate characterization of covariate dependencies in heterogeneous populations. Combining this model with a tailored differential abundance (DA) test leads to a novel DA testing scheme, cosmoDA, that can reduce the false positive detection rate caused by correlated features. cosmoDA uses penalized generalized score matching for parsimonious model fitting. We show on simulated benchmarks that cosmoDA can accurately estimate feature interactions in the presence of population heterogeneity and significantly reduces the false discovery rate when testing for differential abundance of correlated features. Using single‐cell and amplicon data, we illustrate cosmoDA's ability to estimate data‐adaptive Box–Cox‐type data transformations and assess the impact of zero replacement and power transformations on downstream differential abundance results. cosmoDA is available at https://github.com/bio‐datascience/cosmoDA.

## 1 Introduction

Abundance matrices, detailing the compositional makeup of cellular constituents in a sample, are an important data modality derived from modern high‐throughput sequencing (HTS) experiments, including amplicon sequencing [1, 2] and single‐cell RNA‐Sequencing (scRNA‐Seq) [3, 4]. These matrices commonly have the form $\tilde{\text{X}}\in {\mathbb{N}}_{0}^{n\times p}$ and show the abundance of p features (cell types or microbial taxa) in n tissues [5], bacterial communities [6], or microbial habitats [7]. Because the sequencing capacity of HTS experiments is technically limited, each sample only represents a small part of a larger population, rendering the sum of counts in a row non‐quantitative and making the data compositional [8]. Dividing each sample by its total sum yields relative abundance data, which is proportionally equivalent to the original data and constrained to the (p−1)‐dimensional probability simplex [9]:

(1) $$\Delta \equiv \Delta_{p-1}=\left\{x\in {\mathbb{R}}^{p}:x⪰0,\;1_{p}^{⊤}x=1\right\}.$$

Generative models for HTS‐derived compositional data commonly respect compositionality either by transforming the data into Euclidean space through log‐ratio or similar transformations [10, 11], or by using distributions directly defined on the probability simplex. The Dirichlet distribution is a popular choice due to its relatively simple structure and interpretability [3, 12, 13, 14]. The assumption of independent features (apart from the compositional effect) is, however, a major limitation of the Dirichlet distribution. To allow for more complex dependency structures, [15] proposed the class of logistic normal distributions, which include the estimation of feature‐feature interactions. Several lines of research make successful use of logistic normal models (and extensions thereof) for HTS data (see, e.g., [16, 17]) and address the computational challenges in scaling parameter inference to large‐scale datasets [18].

Another challenge in generative HTS data modeling is the presence of zeroes. Since the logistic normal distribution requires the underlying data to be positive due to logarithmic transformations, zero entries in the primary data need to be replaced by positive values [19, 20]. Any such procedure inevitably distorts the measured data compositions, especially for rare features with many zero entries [19], resulting in another source of modeling inaccuracy.

In his seminal work, John Aitchison [21] provided a general class of distributions, the $A^{p-1}$ class, that includes the logistic normal and the Dirichlet distribution as special cases. This class forms the basis for more recent models that extend the $A^{p-1}$ class and do not require zero imputation. For low‐dimensional data, [22] and [23] introduced the polynomially tilted pairwise interaction (PPI) model, which has properties similar to the Dirichlet distribution at the boundaries of the simplex. The class of a‐b power interaction models, introduced by [24], achieves validity on the simplex boundaries by replacing the logarithm with power transformations. These works further use score matching estimation [25] for computationally efficient parameter inference, reducing the estimation problem to solving a (regularized) quadratic optimization problem. However, both the PPI and the a‐b power interaction model currently only allow to model a homogeneous sample population and cannot describe the differences between groups of samples.

**FIGURE 1:**
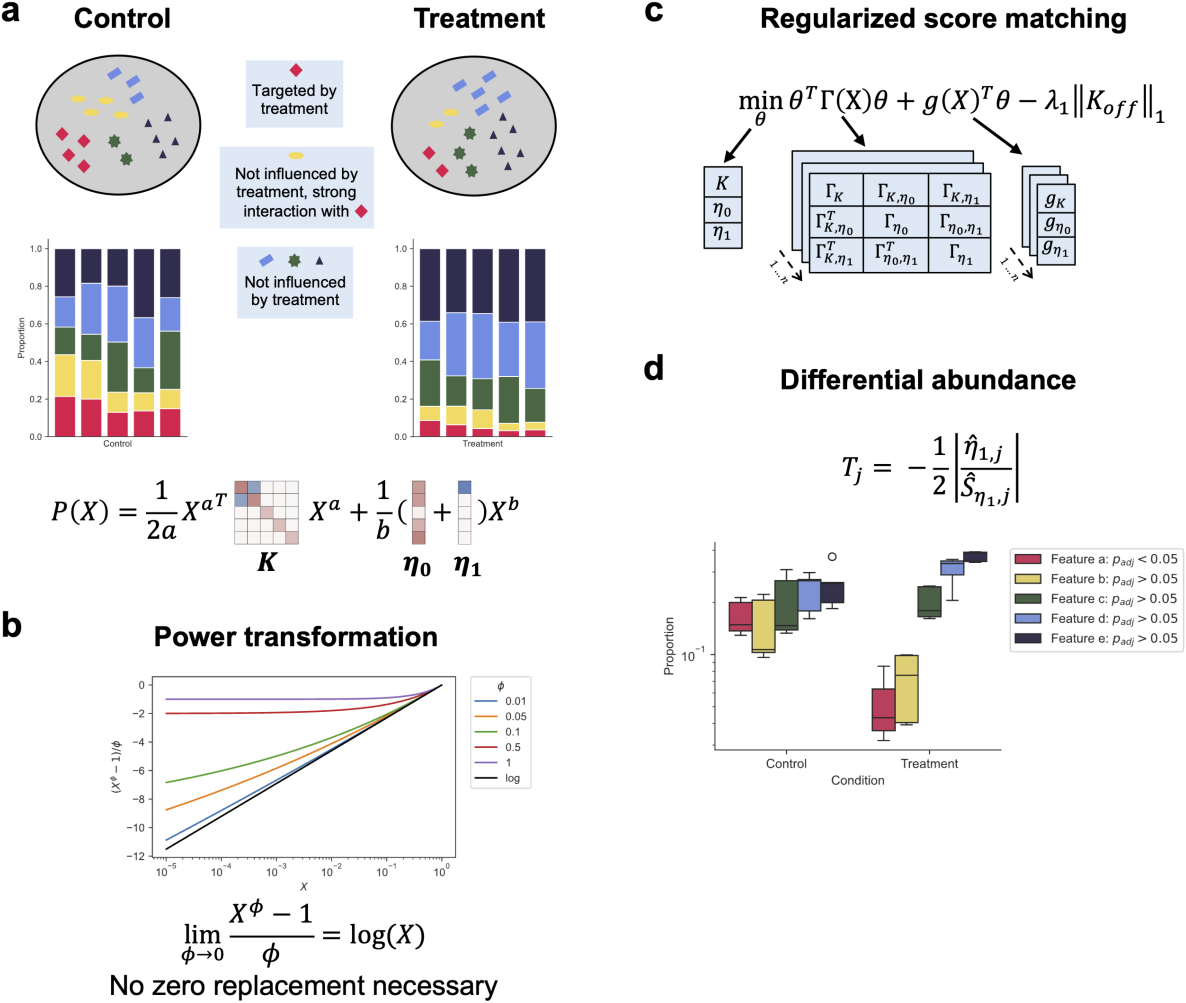
**cosmoDA allows to perform generative modeling and differential abundance testing on compositional data with feature interactions.** (a) Interactions between features can alter the abundance of features although they are not directly associated with the condition. cosmoDA is able to accurately distinguish primary from secondary effects by inferring pairwise feature interactions in addition to the effects associated with the condition. (b) Power transformations allow to analyze compositional data without the imputation of zero values. For decreasing exponents, the Box–Cox transformation converges to the logarithm. (c) cosmoDA uses regularized score matching for parameter inference. The optimization problem, therefore, reduces to a quadratic function with parameters Γ and g defined by averaging over all samples. (d) Differential abundance testing in cosmoDA uses a studentized test statistic. Only the feature primarily associated with the condition (Feature a) has a small adjusted *p*‐value.

A central task in HTS data analysis is the detection of significant differences in the feature composition, given environmental, clinical, or host‐specific perturbations or variations. This problem, also known as differential abundance (DA) testing, faces the same challenges as generative modeling [2, 8]. While compositionality and zero handling are discussed in most state‐of‐the‐art DA testing methods [26, 27, 28], only a few methods explicitly include interactions between compositional features in their testing procedure [29]. Such interactions, however, may contribute to the false discovery of certain features that are not directly impacted by the perturbations or covariate changes, but simply strongly correlate with the differentially abundant feature. Consider a composition of five microbial taxa a,b,c,d,e, where a and b have a symbiotic relationship and their abundances are highly correlated (Figure 1a). A treatment now targets taxon a and causes a decline in its population. This will, in turn, cause the abundance of taxon b to also decrease, although it was not directly influenced by the treatment. Classical DA testing methods will not be able to discern between these primary and secondary effects caused by the treatment, detecting both a and b as differentially abundant.

In this work, we present a new DA framework, termed cosmoDA (**co**mpositional **s**core **m**atching **o**ptimization for **D**ifferential **A**bundance analysis), that addresses the challenge of feature interactions in DA testing. cosmoDA is based on the a‐b power interaction models from Reference [24] and introduces a linear covariate effect on the location vector, thus enabling the inclusion of sample group indicators or continuous covariates of interest. We provide a framework for assessing the significance of the estimated covariate effects, which, in the case of group indicator variables, allows principled compositional differential abundance testing. A similar covariate‐extended model was introduced for low‐dimensional compositional data by [30], albeit only for the special case of the logistic normal model. In the a‐b power interaction models, maximum likelihood estimation is not possible due to the intractability of computing the normalizing constant. We thus resort to the score matching framework [25]. By carefully studying the structural properties of the underlying score matching objective, our extended estimation framework retains the favorable quadratic nature of the underlying optimization problem with negligible computational overhead. Regularization on the interaction effects further ensures model identifiability and selection of the most important correlation patterns. The characteristics of the a‐b power interaction model thus ensure that feature interactions are adequately considered and zero entries in the underlying data do not need to be replaced or imputed.

The remainder of the paper is structured as follows. In the next section, we introduce cosmoDA as an extension of the a‐b power interaction model describes the score matching estimation framework and explains how the power transformation makes zero replacement obsolete. We then describe the model regularization framework, sketch the computational implementation, and introduce the differential abundance testing framework cosmoDA. Section 3 provides several simulated data benchmarks that showcase the ability of cosmoDA to (i) correctly estimate sparse interaction matrices in the presence of covariates and (ii) reduce the false discovery rate in differential abundance testing compared to other state‐of‐the‐art methods. We investigate the impact of different power transformations on differential abundance in real scRNA‐seq and 16S rRNA sequencing data in Section 4 and provide a data‐driven method to select the power exponents in practice. Section 4 discusses the results, highlights strengths and limitations of the work, and provides guidelines for future research. cosmoDA is available as a Python package at https://github.com/bio‐datascience/cosmoDA.

## 2 Methods

We consider to model compositional matrices X where each row $x^{\left(i\right)}\in \Delta$, i=1,…,n, represents a sample, and each column $x_{j}$, j=1,…,p, represents the jth compositional feature. We are motivated by the large number of available biological “compositional” count matrices $\tilde{X}\in {\mathbb{N}}_{0}^{n\times p}$ derived from HTS experiments. Important instances include 16S rRNA amplicon sequencing data, where each feature represents read counts associated with a microbial taxon [8] and scRNA‐Seq experiments, where each feature represents a certain cell‐type proportion, as derived from clustered transcriptional profiles [3, 4]. Due to the compositional nature of the derived count data, a common approach is to scale each observation ${\tilde{x}}^{\left(i\right)}$ by its library size $S^{\left(i\right)}=\sum_{j=1}^{p}x_{j}^{\left(i\right)}$ to obtain relative abundance samples $x^{\left(i\right)}={\tilde{x}}^{\left(i\right)}/S^{\left(i\right)}\in \Delta$ [8].

### 2.1 The Covariate‐Extended A‐B Power Interaction Model

Following the proposal in Reference [24], we model samples in X through the *a‐b power interaction model* on the (p−1)‐dimensional simplex Δ. The unnormalized probability density for one sample $x\equiv x^{\left(i\right)}$ reads:

(2) $$\begin{array}{ll} & p_{\eta ,K}(x)\propto \exp\left(-\frac{1}{2a}{x^{a}}^{⊤}Kx^{a}+\frac{1}{b}\eta^{⊤}x^{b}\right),\\ & x\in \Delta ;\;K=K^{T};\;K1_{p}=0_{p}. \end{array}$$

Here, interactions between features are modeled through the *interaction matrix* $K\in {\mathbb{R}}^{p\times p}$, and the *location vector* $\eta \in {\mathbb{R}}^{p}$ describes the base composition of the individual features. This model belongs to the class of exponential family models. Using the conventions in Reference [24], $x^{a}\equiv \log(x);1/a\equiv 1$ if a=0, and $x^{b}\equiv \log(x);1/b\equiv 1$ if b=0, power interaction models encapsulate several probability distributions as special cases.

With parameter settings a=b=0, the model includes the Dirichlet distribution with the additional constraints K=0,η≻−1, the logistic normal distribution [15] with the constraints $K1_{p}=0_{p},x^{T}Kx>0\;\forall x,1_{p}^{T}\eta =-p$, and Aitchison's $A^{p-1}$ family of distributions [21] with $x^{T}Kx>0\;\forall x,\eta ⪰-1$. For the logistic normal case, the interaction matrix K is equivalent to the inverse covariance matrix of logratio‐transformed data, given specific linear transformations [31]. With parameter settings a=1 and b=0, the model is equivalent to the PPI distribution [22, 23] (see Appendix A), and with parameter settings a=b=1, the power interaction model is equivalent to the maximum entropy distribution on the simplex with the constraints $K1_{p}=0_{p},x^{T}Kx>0\;\forall x$, as derived in Reference [32].

As stated in Theorem 1 from Reference [24], the probability density in Equation (2) is proper if either

- a>0,b>0;
- $a>0,\;b=0,\;\eta_{j}>-1\;\forall j$;
- $a=0,\;b=0,\;\log{\left(x\right)}^{T}K\log(x)>0\;\forall x\in \Delta$;
- $a=0,\;b>0,\;\log{\left(x\right)}^{T}K\log(x)\geq 0\;\forall x\in \Delta$.

We next extend the original proposal of the a‐b power interaction model by including a (continuous or binary) covariate vector $y\in {\mathbb{R}}^{n}$ (or $y\in {\left\{0,1\right\}}^{n}$, respectively) in the model. The covariate describes, for example, a concurrently measured quantity of interest for each sample, or, more relevant in our context, a condition‐specific indicator vector. We model the influence of $y\equiv y^{\left(i\right)}$ on $x^{\left(i\right)}$ by introducing a linear model on the location vector η:

(3) $$\eta =\eta_{0}+y\eta_{1}.$$

Plugging this model into Equation (2) results in the covariate‐extended a‐b power interaction model:

(4) $$\begin{array}{ll} & p_{\eta ,K}(x)\propto \exp\left(-\frac{1}{2a}{x^{a}}^{⊤}Kx^{a}+\frac{1}{b}{\left(\eta_{0}+y\eta_{1}\right)}^{⊤}x^{b}\right),\\ & x\in \Delta ;\;K1_{p}=K^{T}1_{p}=0_{p}. \end{array}$$

This formulation of the model assumes that all samples stem from an overall population with fixed interaction matrix K, but allows the proportions of features, described by η, to be dependent on the measured covariate y. For the probability density of the covariate‐extended a‐b power interaction model to be proper, the same conditions hold as for the model in Equation (2), replacing η with $\eta_{0}+y\eta_{1}$.

The model in Equation (4) forms the basis for our differential abundance testing framework cosmoDA (**co**mpositional **s**core **m**atching **o**ptimization for **D**ifferential **A**bundance analysis). In case where y represents a binary group indicator, for example, case versus control samples, cosmoDA fits the data to the model and tests for significant changes of the individual components of $\eta_{1}$. Figure 1 provides a conceptual overview of cosmoDA. Before detailing the specific test statistics, we describe the underlying parameter estimation framework.

### 2.2 Model Estimation

#### 2.2.1 Score Matching for A‐B Power Interaction Models

Efficient parameter estimation for the a‐b power interaction models Equation (2) through generalized score matching [25, 33] was proposed by [24]. While other estimation approaches (e.g., pseudo‐likelihood estimation as used by [32] in maximum‐entropy models) might also provide efficient parameter estimation for our model, [34] and [35] showed score matching to be preferential for similar distributions from the exponential family. Given an (unknown) true data distribution $P_{0}$ with density $p_{0}$ and a family of distributions of interest 𝒫(𝒟), score matching tries to find P∈𝒫(𝒟) with density p such that the Hyvärinen divergence between the gradients of the logarithm of the densities of $P_{0}$ and P is minimized:

(5) $$\frac{1}{2}\int_{𝒟}p_{0}(x)\|\nabla \log p(x)⊙{\tilde{h}}^{1/2}(x)-\nabla \log p_{0}(x)⊙{\tilde{h}}^{1/2}(x){\|}_{2}^{2}dx,$$

where $\tilde{h}(x)=({\tilde{h}}_{1}(x_{1}),\ldots ,{\tilde{h}}_{p}(x_{p}))$ is a weight function. Yu et al. [36] show that score matching can be performed on domains with positive Lebesgue measure in ${\mathbb{R}}^{p}$ by setting $\tilde{h}$ such that $\nabla \log p(x)⊙{\tilde{h}}^{1/2}(x)$ does not vanish at the boundaries of the domain.

Yu et al. [24] adapted the generalized score matching framework for the a‐b power interaction models on the (p−1)‐dimensional probability simplex in ${\mathbb{R}}^{p}$ by profiling out the last coordinate $x_{p}\equiv 1-\sum_{j=1}^{m-1}x_{j}$, similar to the additive log‐ratio transformation. We follow this approach, setting ${\tilde{h}}_{j}(x)=(h_{j}\circ \varphi_{j})(x)$ with $h_{j}(x)=x_{j}^{c}$ and $\varphi_{j}(x)=\min\{x_{j},x_{p},C_{j}\}$ and fixing $C_{j}=1$ and c=2, as recommended by [24]. This results in a weight function ${\tilde{h}}_{j}(x)=\min{\left\{x_{j},x_{p}\right\}}^{2}$. With p(x) from the family of a‐b power interaction models, the following mild assumptions hold [24]:

1. $p_{0}(x_{j};x_{-j})h_{j}(\varphi {\left(x\right)}_{j})\partial_{j}\log p{\left.\left(x_{j};x_{-j}\right)\right|}_{x_{j}↘a_{k}{\left(x_{-j}\right)}^{+}}^{x_{j}↗b_{k}{\left(x_{-j}\right)}^{-}}=0$ for all $k=1,\ldots ,K_{j}(x_{-j})$ and $x_{-j}\in {𝒮}_{-j,𝒟}$ for all j=1,…,p;
2. $\int_{𝒟}p_{0}(x){\left\|\nabla \log p(x)⊙{\left(h\circ \varphi \right)}^{1/2}(x)\right\|}_{2}^{2}dx<+\infty$, $\int_{𝒟}p_{0}(x){\left\|{\left[\nabla \log p(x)⊙(h\circ \varphi )(x)\right]}^{\prime }\right\|}_{1}dx<+\infty$.
3. ∀j=1,…,p and almost everywhere $x_{-j}\in {𝒮}_{-j,𝒟},$, the component function $h_{j}$ of h is absolutely continuous in any bounded sub‐interval of the section ${𝒞}_{j,𝒟}(x_{-j})$.

Therefore, a consistent estimator of the loss function Equation (5) follows as a sample‐ and feature‐wise sum over the entire dataset:

(6) $$\begin{array}{ll} {\hat{L}}_{h}(P) & =\frac{1}{2}\overset{p}{\underset{j=1}{\sum }}\overset{n}{\underset{i=1}{\sum }}\frac{1}{2}(h_{j}\circ \varphi_{j})\left(X^{\left(i\right)}\right)\cdot {\left[\partial_{j}\log p\left(X^{\left(i\right)}\right)\right]}^{2}\\ & \;+\partial_{j}\left[(h_{j}\circ \varphi_{j})\left(X^{\left(i\right)}\right)\cdot \partial_{j}\log p\left(X^{\left(i\right)}\right)\right], \end{array}$$

where $X^{\left(i\right)}$, 1≤i≤n, form an i.i.d. sample from the unknown data distribution $P_{0}$ and P is an a‐b power interaction model with unnormalized density as described in Equation (2). Aggregating K and η to θ=(vec(K),η) and defining $P_{\theta }$ and its density $p_{\theta }$ accordingly shows that the power interaction model without covariate Equation (2) follows an exponential‐family‐type model

(7) $$\log p_{\theta }(x)=\theta^{⊤}t(x)-\psi (\theta )+b(x),x\in \Delta ,$$

where the function t(·) denotes the function for the sufficient statistics, ψ(·) the cumulant function, and b(·) the logarithm of the base measure, respectively.

Then, ${\hat{L}}_{h}$ can be reformulated as a quadratic optimization problem:

(8) $${\hat{L}}_{h}(P_{\theta })=\frac{1}{2}\theta^{⊤}\Gamma (x)\theta -g{\left(x\right)}^{⊤}\theta +\mathrm{const}.$$

with $\Gamma (x)\in {\mathbb{R}}^{r\times r}$ and $g(x)\in {\mathbb{R}}^{r}$ are sample averages of known functions in x only.

Analogously, the same considerations hold for the covariate‐extended a‐b power interaction model Equation (4), substituting η with $\eta_{0}+y\eta_{1}$. This substitution does not yet provide individual estimates of $\eta_{1}$ and $\eta_{0}$, though, which are required for differential abundance testing. To obtain these individual estimates, a look at the exact derivation of Γ and g, as described by [24], is necessary. We first split the location vector into its two parts $\eta_{0}$ and $\eta_{1}$, and set $\theta =(\text{vec}(K),\eta_{0},\eta_{1})$. After dropping the last coordinate by assuming $x_{p}\equiv 1-1_{p-1}^{⊤}x_{-p}$ as above, the first and second partial derivatives for the covariate‐less model (Equations (4.1) and (4.2) in Reference [24]) can easily be adapted to the covariate‐extended model:

(9) $$\begin{array}{ll} \partial_{j}\log p(x_{-p}) & =-\left(\kappa_{,j}^{⊤}x^{a}\right)x_{j}^{a-1}+\left(\kappa_{,p}^{⊤}x^{a}\right)x_{p}^{a-1}+\eta_{j}x_{j}^{b-1}-\eta_{p}x_{p}^{b-1},\\ & =-\left(\kappa_{,j}^{⊤}x^{a}\right)x_{j}^{a-1}+\left(\kappa_{,p}^{⊤}x^{a}\right)x_{p}^{a-1}\\ & \;+\eta_{0,j}x_{j}^{b-1}-\eta_{0,p}x_{p}^{b-1}+y_{j}\eta_{1,j}x_{j}^{b-1}-y_{p}\eta_{1,p}x_{p}^{b-1} \end{array}$$

(10) $$\begin{array}{ll} \partial_{jj}\log p(x_{-p}) & =-(a-1)\left[\left(\kappa_{,j}^{⊤}x^{a}\right)x_{j}^{a-2}+\left(\kappa_{,p}^{⊤}x^{a}\right)x_{p}^{a-2}\right]\\ & \;-a\left[\kappa_{jj}x_{j}^{2a-2}+\kappa_{pp}x_{p}^{2a-2}+2\kappa_{jp}x_{j}^{a-1}x_{p}^{a-1}\right]\\ & \;+(b-1)\left[\eta_{j}x_{j}^{b-2}+\eta_{p}x_{p}^{b-2}\right]\\ & =-(a-1)\left[\left(\kappa_{,j}^{⊤}x^{a}\right)x_{j}^{a-2}+\left(\kappa_{,p}^{⊤}x^{a}\right)x_{p}^{a-2}\right]\\ & \;-a\left[\kappa_{jj}x_{j}^{2a-2}+\kappa_{pp}x_{p}^{2a-2}+2\kappa_{jp}x_{j}^{a-1}x_{p}^{a-1}\right]\\ & \;+(b-1)\left[\eta_{0,j}x_{j}^{b-2}+\eta_{0,p}x_{p}^{b-2}\right]\\ & \;+(b-1)\left[y_{j}\eta_{1,j}x_{j}^{b-2}+y_{p}\eta_{1,p}x_{p}^{b-2}\right] \end{array}$$

Plugging these definitions into the loss function in Equation (6) and rearranging the individual terms in the same way as in Reference [24] yields Γ and g as follows (Figure 1c):

(11) $$\begin{array}{ll} & \Gamma \equiv \left[\begin{array}{ccc} \Gamma_{K} & \Gamma_{K,\eta_{0}} & \Gamma_{K,\eta_{1}}\\ \Gamma_{K,\eta_{0}}^{⊤} & \Gamma_{\eta_{0}} & \Gamma_{\eta_{0},\eta_{1}}\\ \Gamma_{K,\eta_{1}}^{⊤} & \Gamma_{\eta_{0},\eta_{1}}^{⊤} & \Gamma_{\eta_{1}} \end{array}\right]\in {\mathbb{R}}^{\left(p^{2}+2p)\times (p^{2}+2p\right)},\\ & g\equiv \left(\text{vec}(g_{K}),\;g_{\eta_{0}},\;g_{\eta_{1}}\right)\in {\mathbb{R}}^{p^{2}+2p}, \end{array}$$

where Γ and g have a block structure with $\Gamma_{K}\in {\mathbb{R}}^{p^{2}\times p^{2}}$, $\Gamma_{K,\eta_{0}}\in {\mathbb{R}}^{p^{2}\times p}$, $\Gamma_{K,\eta_{1}}\in {\mathbb{R}}^{p^{2}\times p}$, $\Gamma_{\eta_{0}}\in {\mathbb{R}}^{p\times p}$, $\Gamma_{\eta_{0},\eta_{1}}\in {\mathbb{R}}^{p\times p}$, $\Gamma_{\eta_{1}}\in {\mathbb{R}}^{p\times p}$, and $g_{K}\in {\mathbb{R}}^{p^{2}}$, $g_{\eta_{0}}\in {\mathbb{R}}^{p}$, $g_{\eta_{1}}\in {\mathbb{R}}^{p}$.

The exact derivations are shown in Appendix B. By recognizing that each entry of Γ and g can be written as a mean over all samples, the elements related to $\eta_{1}$ can be computed directly from the elements related to $\eta_{0}$:

$$\begin{array}{ll} \Gamma_{K,\eta_{1}} & =\frac{1}{n}\overset{n}{\underset{i=1}{\sum }}y\Gamma_{K,\eta_{0}}^{\left(i\right)}\\ \Gamma_{\eta_{0},\eta_{1}} & =\frac{1}{n}\overset{n}{\underset{i=1}{\sum }}y\Gamma_{\eta_{0}}^{\left(i\right)}\\ \Gamma_{\eta_{1}} & =\frac{1}{n}\overset{n}{\underset{i=1}{\sum }}y^{2}\Gamma_{\eta_{0}}^{\left(i\right)}\\ g_{\eta_{1}} & =\frac{1}{n}\overset{n}{\underset{i=1}{\sum }}yg_{\eta_{0}}^{\left(i\right)}. \end{array}$$

Therefore, the computational overhead for computing the additional sub‐matrices and sub‐vectors related to $\eta_{1}$ is negligible. Still, the addition of p dimensions to the optimization problem Equation (8) increases the problem dimensionality from $p^{2}+p$ to $p^{2}+2p$ compared to the covariate‐less model.

#### 2.2.2 Model Identifiability Through Regularization

Since the number of parameters in the power interaction model scales quadratically with p, real HTS data applications are in the high‐dimensional regime with more parameters than samples, that is, $p^{2}+2p>n$. To enable model identification, we place a $\ell_{1}$ regularization penalty on the off‐diagonal elements $K_{\text{off}}$ of K:

(12) $${\hat{L}}_{h,C,\lambda_{1},\delta }(P_{\theta })=\frac{1}{2}\theta^{⊤}\Gamma_{\delta }(x)\theta -g{\left(x\right)}^{⊤}\theta +\lambda_{1}\|\text{vec}(K_{\text{off}}){\|}_{1}.$$

As defined in Section 2.2.1, $\theta =(\text{vec}(K),\eta_{0},\eta_{1})$ comprises all model parameters, and $P_{\theta }$ denotes the power interaction model with probability density $p_{\theta }$ defined in Equation (4). Following [24], we multiply the diagonal entries of Γ(x) corresponding to K by a factor δ>1 to avoid an unbounded loss function. We denote Γ(x) with scaled diagonal entries as $\Gamma_{\delta }(x)$. Here, we use the default value from the implementation of [24], $\delta =2-\frac{1}{1+4e\max(6\log(p)/n,\sqrt{6\log(p)/n})}$. In cases where p≫n, the entries of $\eta_{0}$ and $\eta_{1}$ can be penalized as well with a regularization parameter $\lambda_{2}$:

(13) $$\begin{array}{ll} & {\hat{L}}_{h,C,\lambda_{1},\lambda_{2},\delta }(P_{\theta })=\frac{1}{2}\theta^{⊤}\Gamma_{\delta }(x)\theta -g{\left(x\right)}^{⊤}\theta +\lambda_{1}\|\text{vec}(K_{\text{off}}){\|}_{1}\\ & \;+\lambda_{2}\|\eta_{0}{\|}_{1}+\lambda_{2}\|\eta_{1}{\|}_{1} \end{array}$$

Furthermore, assuming K to be sparse matches the widely popular view of sparse association networks between microbial features or cell types (see, e.g., [37]). In the following, we will focus our attention on models without regularization on the location parameter.

Theorem 4 in Reference [24] details that for a proper a‐b power interaction model (see Section 2.1), a boundary function ${\tilde{h}}_{j}(x)$ as described in Section 2.2.1, and under some mild conditions to the regularization strength λ and diagonal multiplier δ, the estimation error is bounded closely to the true values of K and η. As our extension only divides η into two components $\eta_{0}$ and $\eta_{1}$, similar guarantees hold for the covariate‐extended a‐b power interaction model.

ALGORITHM 1

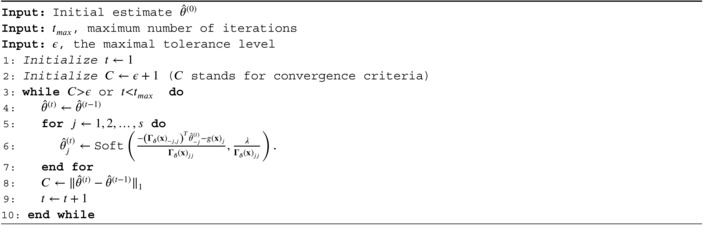

#### 2.2.3 Computational Implementation

The regularized score matching loss ${\hat{L}}_{h,C,\lambda ,\delta }(P_{\theta })$ Equation (12) represents a (large‐scale) $\ell_{1}$‐penalized quadratic optimization problem that can be numerically solved with a variety of optimization methods. Here, we follow [24] and employ a proximal coordinate descent scheme (see also Algorithm 2 in Reference [35]). This algorithm also covers the covariate‐extended a‐b power interaction model and is described in Algorithm 1. Here, s is the dimensionality of θ and Soft(·) is the softmax function. The default settings in cosmoDA are $\epsilon =10^{-1}$ and $t_{max}=1000$.

At its core, our Python implementation of Algorithm 1 uses the C implementation from the *genscore* package [24, 34] and included in the cosmoDA Python package. The cosmoDA package also provides an interface for a‐b power interaction models that is equivalent to the R interface in the *genscore* package.

### 2.3 Differential Abundance Testing

One of the key objectives of cosmoDA is to determine the statistical significance of the covariate effects $\eta_{1,j}$ for every feature j=1…p. Here, we combine results from References [27] and [22] to test the null hypothesis

$$H_{0}:\eta_{1,j}=0\;\text{against the alternative}\;H_{1}:\eta_{1,j}\neq 0.$$

Let $\hat{\theta }=(\text{vec}(\hat{K}),{\hat{\eta }}_{0},{\hat{\eta }}_{1})$ be the parameter estimates obtained from the score matching estimation framework. Scealy and Wood [22] show that, under certain technical conditions and assumptions (see Theorems 1 and 2 in Reference [22]) the quantity $\hat{S}=\Gamma^{-1}(x){\hat{\sum }}_{0}\Gamma^{-1}(x)$ yields a consistent estimator for $\mathrm{Var}(\hat{\theta })$. In cosmoDA, we estimate ${\hat{\sum }}_{0}$ as follows:

(14) $${\hat{\sum }}_{0}=\frac{1}{n}\overset{n}{\underset{i=1}{\sum }}({\tilde{\Gamma }}_{\delta }^{\left(i\right)}(x)\hat{\theta }-{\tilde{g}}^{\left(i\right)}(x)){\left({\tilde{\Gamma }}_{\delta }^{\left(i\right)}(x)\hat{\theta }-{\tilde{g}}^{\left(i\right)}(x)\right)}^{T},$$

where ${\tilde{\Gamma }}_{\delta }^{\left(i\right)}(x)$ and ${\tilde{g}}^{\left(i\right)}(x)$ are the components of ${\tilde{\Gamma }}_{\delta }(x)$ and g(x) corresponding to the i‐th sample. By selecting the components of $\hat{S}$ corresponding to $\eta_{1,j}$, we derive the studentized test statistic

(15) $$T_{j}={\hat{\eta }}_{1,j}/{Ŝ}_{\eta_{1,j}},$$

which approximately follows a *t*‐distribution with n−3 degrees of freedom. The corresponding asymptotic *p*‐values read:

(16) $$p_{j}=2F_{t,n-3}(-|T_{j}|),$$

where $F_{t,n-3}$ is the cumulative distribution function of the *t*‐distribution with n−3 degrees of freedom [27]. In cosmoDA, raw *p*‐values are adjusted for multiple testing using the Benjamini–Hochberg procedure [38] (see Figure 1d for illustration).

We empirically verified the distribution of test statistics in Figure F1 with the help of data from our model comparison benchmark with p=11 (Section 3.2). There, we plotted the distribution of test statistics for features following the null hypothesis (i.e., without differential abundance) for both simulated sample sizes n=100 and n=1000. These distributions closely match the densities of the respective $t_{n-3}$‐distributions.

### 2.4 Model Selection and Hyperparameter Tuning

To make cosmoDA fully data‐adaptive, we provide several strategies to select the hyperparameters of the framework. We first describe regularization parameter selection, followed by a novel data‐driven approach to select the exponents a and b in a‐b power interaction models.

#### 2.4.1 Regularization Parameter Selection

cosmoDA provides several model selection methods to determine the regularization parameter $\lambda_{1}$ (and $\lambda_{2}$, respectively). The default strategy is k‐fold cross‐validation (k=5) with the 1SE rule [39]. Here, the largest $\lambda_{1}$ value is chosen that lies within one standard error band of the $\lambda_{1}$ that minimizes the cross‐validated regularized score matching loss Equation (12). The range of the $\lambda_{1}$‐path is chosen to cover the whole range of possible sparsity of K, that is, from a fully dense K to a diagonal K (see Figure F16b). This range depends on the dimensions of the dataset at hand and the chosen power transformation (see Appendix C). Per default, our implementation considers 100 $\lambda_{1}$‐values log‐linearly spaced in the interval $\left[10^{-6},1\right]$.

cosmoDA also allows $\lambda_{1}$ to be selected via the extended Bayesian Information Criterion (eBIC, [40]). Following [34], the eBIC for the a‐b power interaction model reads:

(17) $$\begin{array}{ll} & {\text{eBIC}}_{\gamma }(\text{vec}(K_{\text{off}}))=S(\text{vec}(K_{\text{off}}))\log(n)-2\log({\hat{L}}_{h,C,\lambda_{1},\delta }(P_{\theta })\\ & \;+2\gamma \|\text{vec}(K_{\text{off}}){\|}_{1}, \end{array}$$

where $S(\text{vec}(K_{\text{off}}))$ denotes the size of the support of $\text{vec}(K_{\text{off}})$. The default γ value is γ=0.5.

#### 2.4.2 Data‐Driven Selection of A‐B Powers

A key strength of a‐b power interaction models is their seamless applicability to compositional data with excess zeros. While the limiting case a=b=0 (i.e., log‐transforming the data) requires a strategy for zero replacement or zero imputation [19, 20] with potentially detrimental effects for downstream analysis [41], we propose a data‐driven tuning strategy for power interaction models with powers a>0 and b>0 that keeps the original data unaltered. For simplicity, we consider the setting a=b.

We first note that the power transformations in the models (2) and (4) are similar to the Box–Cox transformation [42] of the form:

(18) $$x_{\phi }=\left\{\begin{array}{ll} \frac{1}{\phi }(x^{\phi }-1),\; & \text{if}\;\phi >0\\ \log x,\; & \text{if}\;\phi =0, \end{array}\right.$$

with $\lim_{\phi \to 0}\frac{1}{\phi }(x^{\phi }-1)=\log(x)$ (see also Figure 1b for illustration). The Box–Cox transformation and the power transformation used in a‐b power interaction models Equations (2), (4) are, however, not equivalent due to the −1 term in the Box–Cox transformation. By introducing scaling factors for the score matching elements in Equation (11), we can nevertheless achieve the same asymptotic approximation to the logarithm as the Box–Cox transformation (see Appendix C for details).

While ϕ is typically tuned to make the transformed data approach normality, we follow a geometric strategy inspired by the one presented in Reference [43]. Specifically, we determine ϕ=a=b to let the resulting “geometry” of transformed data be as similar as possible to the appropriate log‐ratio geometry. This is achieved by maximally aligning the principal component (PC) embedding of log‐ratio transformed data with imputed zeros and the PC embedding of a power transformation with parameter ϕ of the data with zero entries [44]. Maximal alignment is defined as the highest Procrustes correlation of both embeddings over a range of values ϕ∈]0,1[. In the case of power interaction models, it is natural to select a power that closely matches the geometry of data after the additive log‐ratio (ALR) transform, since a‐b power interaction models with ϕ=a=b=0 are a generalization of Aitchison's $A^{p-1}$ distributions after ALR transformation of the data [15]. Since equal dimensionality of the ALR and power‐transformed data is required, we append the column $\log(\frac{X_{p}}{X_{p}})=0_{p}$ to the ALR transformation of X before performing PC analysis.

The original procedure to obtain maximal Procrustes correlation is outlined in Reference [43]. We use the same procedure, but with different input matrices. Let $X_{\phi }$ be the Box–Cox‐like transformed data with

(19) $$X_{\phi ,j}=\frac{1}{\phi }\left(p\frac{X_{j}^{\phi }}{\sum_{k=1}^{p}X_{k}^{\phi }}-1\right),$$

and $X_{ALR}$ is the ALR‐transformed data (with pseudocount 0.5 for all zeros) with column $0_{p}$ appended.

We compute the Procrustes correlation $r_{\phi }$ between the two data matrices as follows:

$$\begin{array}{ll} & \text{(i)}\;\;\;\text{Matrix normalization:}\;\;\;X_{\phi }^{*}=X_{\phi }/\sqrt{\text{trace}(X_{\phi }^{T}X_{\phi })}\\ & \;X_{ALR}^{*}=X_{ALR}/\sqrt{\text{trace}(X_{ALR}^{T}X_{ALR})}\\ & \text{(ii)}\;\;\;\text{SVD of cross product:}\;\;\;S={X_{\phi }^{*}}^{T}X_{ALR}^{*}=U\sum V^{T}\\ & \text{(iii)}\;\;\;\text{Optimal rotation matrix:}\;\;\;Q=VU^{T}\\ & \text{(iv)}\;\;\;\text{Sum of squared errors:}\;\;\;E_{\phi }=\text{trace}\\ & \;({\left(X_{\phi }^{*}-X_{ALR}^{*}Q\right)}^{T}(X_{\phi }^{*}-X_{ALR}^{*}Q))\\ & \text{(v)}\;\;\;\text{Procrustes correlation:}\;\;\;r_{\phi }=\sqrt{1-E_{\phi }} \end{array}$$

For a given dataset, the optimal power $\phi^{*}$ is determined by $\phi^{*}:=\arg\max_{\phi }r_{\phi }$ for ϕ∈]0,1[.

## 3 Simulation Benchmarks

We next provide two simulation studies that benchmark two key features of cosmoDA: (i) sparse recovery of feature interactions in the covariate‐extended a‐b power interaction model and (ii) identification of differentially abundant features in the presence of feature correlations. The first benchmark complements the extensive covariate‐free simulation benchmarks of [24], the second one provides a new realistic semi‐synthetic simulation and evaluation setup, incorporating scRNA‐Seq data [45].

### 3.1 Sparse Recovery of Feature Interactions in the Presence of a Covariate

One of the core strengths of a‐b power interaction models is their ability to recover (potentially) sparse feature interaction matrices K. Yu et al. [24] provides an extensive simulation framework that evaluates the influence of hyperparameters, sample size, and interaction topologies on the recovery performance of the a‐b power interaction model. We focus here on evaluating the influence of covariate inclusion on the model's ability to identify sparse feature interactions. Specifically, we expect interaction recovery to be *independent of covariate inclusion*.

Following [24], we generated compositional data $X\in \Delta_{p-1}^{n}$ from an $A^{p-1}$ model with p=100 features using the model in Equation (4) with the constraint that $x^{T}Kx>0\;\forall x,\eta ⪰-1$. To probe sample size dependencies, we used two scenarios: n=80 and n=1000, respectively. We set $\eta_{0}={-1}_{p}$, and considered banded interaction matrices K with bandwidths s=2 if n=80 and s=7 if n=1000, as suggested by [24]. We further defined the nonzero off‐diagonal entries of K as $K_{i,j}=|i-j|/(s+1)-1$ for all i≠j,1≤|i−j|≤s, and the diagonal entries as the negative sum of the off‐diagonals, to ensure the sum‐to‐zero constraint on the rows of K (Figure F2). This definition slightly deviates from the definition in Reference [24], as the sign of all entries in K is flipped, but ensures positive definiteness of K. This modification allows the efficient use of the adaptive rejection sampler for data generation, as provided in the *genscore* R package [34]. For both sample sizes, we generated R=50 replicates of the data.

We applied three different methods for regularized estimation of the underlying interaction matrix K to all datasets:

i. The a‐b power interaction model (a=b=0) without covariate Equation (2). This model allows the estimation of K and $\eta_{0}$. We estimated these models through the implementation in cosmoDA.
ii. The covariate‐extended a‐b power interaction model (a=b=0) Equation (4). Here, we used a misspecified y where each entry is drawn uniformly at random from {0,1}. The model allows the estimation of $K,\eta_{0}$, and $\eta_{1}$. We used the implementation in cosmoDA.
iii. The graphical lasso model on CLR‐transformed data, as introduced in *SPIEC‐EASI* [37]. The non‐zero entries of the resulting sparse inverse covariance matrix serve as a (mis‐specified) proxy for K. We used the implementation from the *gglasso* package [46]. Model selection was performed with the extended BIC (eBIC) criterion [40] with γ=0.25.

For all three models, we used $n_{\lambda }=100$ values for the regularization parameter, log‐spaced in the range $10^{-6}<\lambda_{1}<1$, and k=5 cross‐validation folds. All score matching estimation parameters were set to the defaults recommended by [24] (see also Section 2.2.3).

To measure recovery performance, we compared the support of the off‐diagonal elements of the estimated $\hat{K}$ and the ground truth K by calculating the true positive rate (TPR) and true negative rate (TNR), and assessing them through the receiver operating characteristic (ROC) curves.

**FIGURE 2:**
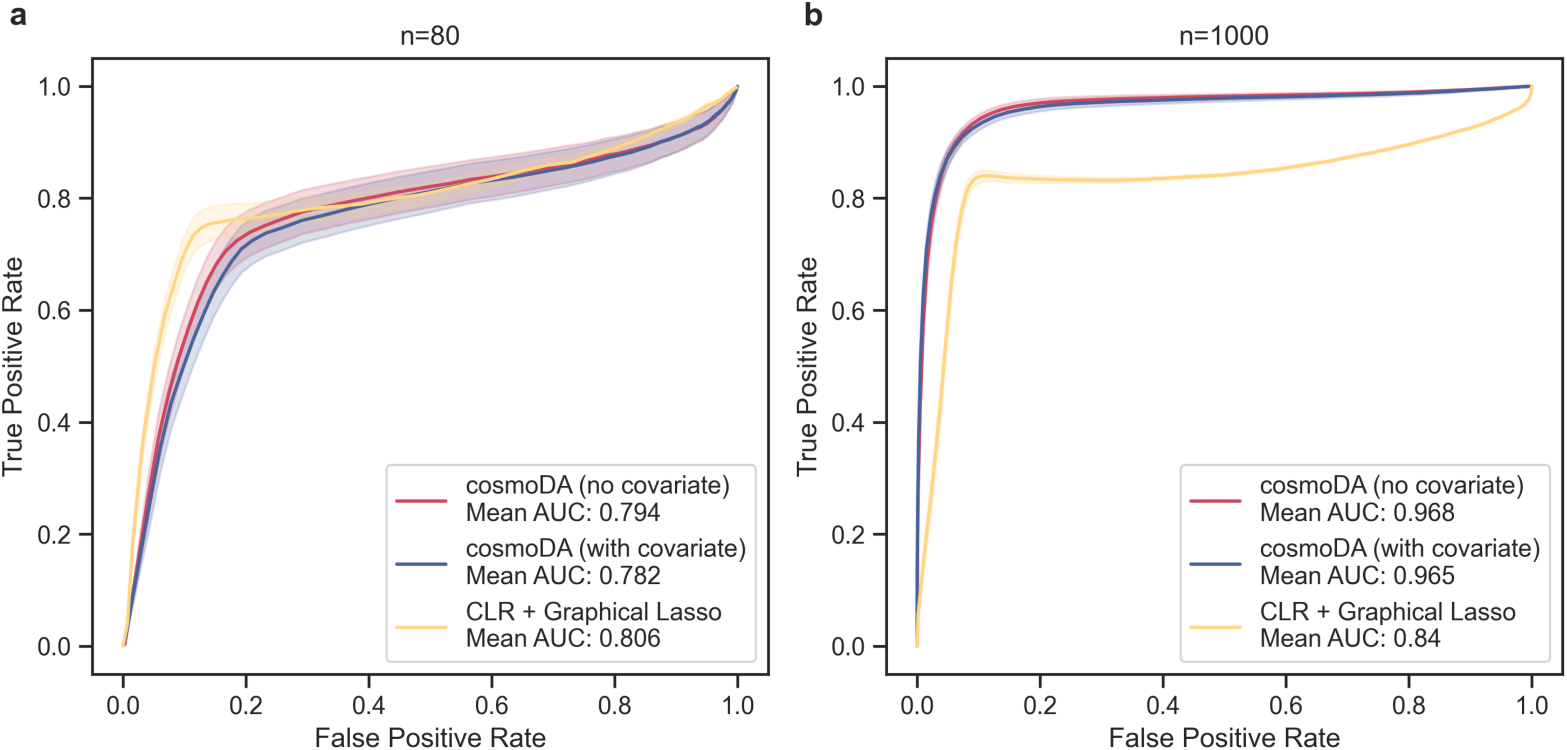
**Recovery of K improves with sample size and is not impacted by covariate inclusion.** Receiver operating curves for cosmoDA with and without covariate effect estimation, as well as CLR transform and graphical lasso for with (a) n=80 and (b) n=1000. The solid lines depict the mean ROC over all 50 generated datasets, and the shaded areas show the standard error.

Figure 2 summarizes the average ROC curves for the two different sample sizes. For n=80 (Figure 2a), we observed that both a‐b power interaction models showed almost equivalent ability to reconstruct the interaction matrix (mean AUC 0.782 vs. 0.794). Their performance was slightly worse than the graphical lasso (mean AUC 0.806), especially for false positive rates smaller than 0.2. When increasing the sample size to n=1000, all three methods showed improvements in recovery performance, improving the mean average AUC as well as reducing the variance in results (Figure 2b). As expected, including a covariate in the a‐b power interaction model had only a marginal impact on the mean AUC (0.965 vs. 0.968). Contrary to the low sample size case, both a‐b power interaction models significantly outperformed the (misspecified) graphical lasso (mean AUC 0.84) across the entire range of regularization strengths.

### 3.2 Differential Abundance Testing in the Presence of Correlated Features

To test the effectiveness of cosmoDA in detecting differentially abundant features in the presence of realistic feature interactions, we designed the following semi‐synthetic simulation benchmark.

**FIGURE 3:**
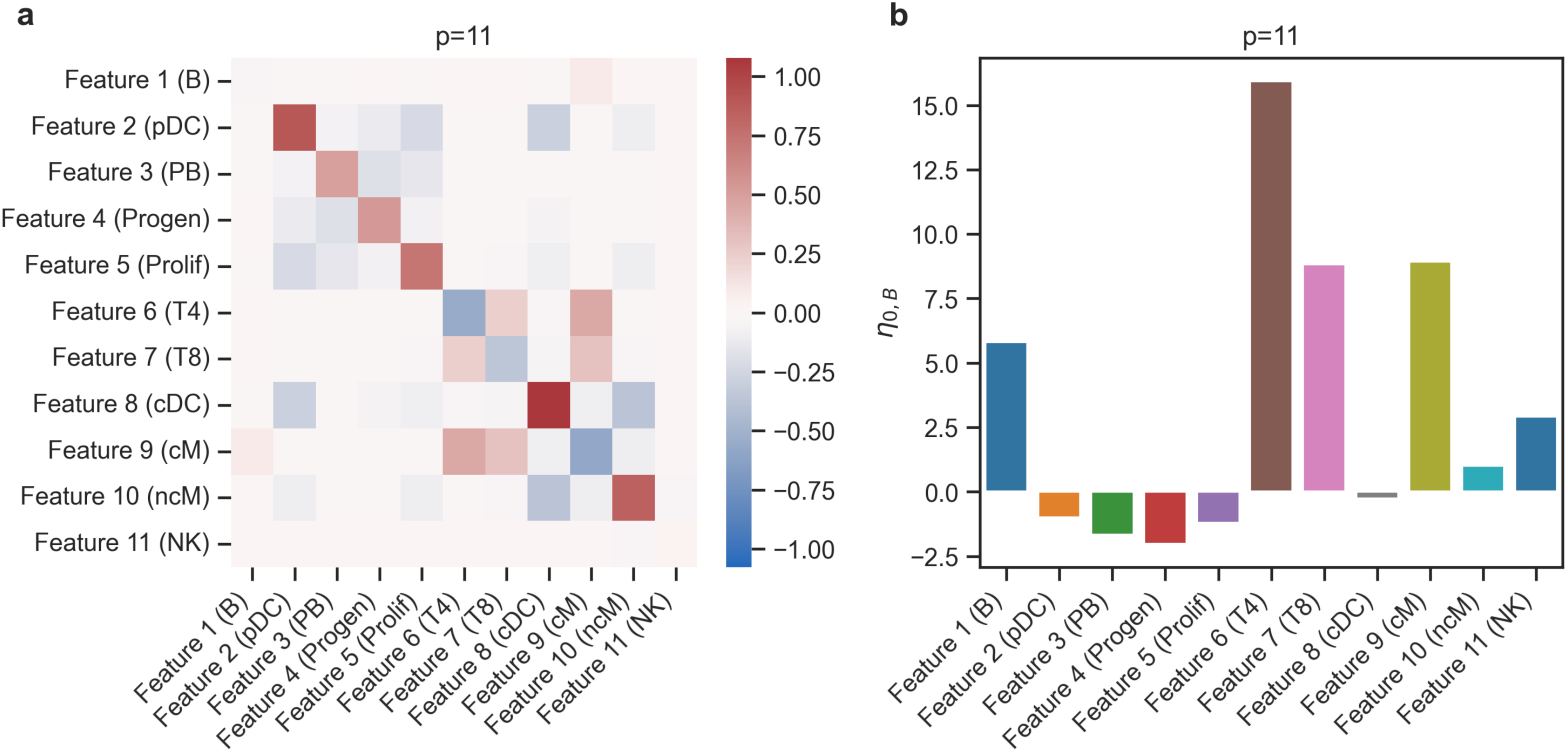
**Data generation parameters used for the Differential abundance testing benchmark,** p=11. Parameters were generated by running the power interaction model without covariate on the dataset from [45]. The names of the cell types from the original dataset are shown in brackets. (a) Interaction matrix ($K_{B}$), (b) Location vector ($\eta_{0,B}$).

We considered a scRNA‐seq data set from Reference [45] that derived relative abundance values of p=11 types of peripheral blood mononuclear cells (PBMCs) from overall n=352 samples. The samples come from 260 unique subjects, 162 of whom are patients with systemic lupus erythematosus (SLE) (208 samples) and 98 healthy controls (144 samples). We used these data to estimate realistic base values for the interaction matrix K and the location vector η, respectively. The base model is the a‐b power interaction model without covariate Equation (2). We set a=b=0 and used $\lambda_{1}=0.043$ as sparsity parameter. We considered the NK cell type as the pth reference component for all power interaction models due to their high abundance and low variance between groups. The resulting interaction matrix $K_{B}$ and location vector $\eta_{0,B}$ are shown in Figure 3.

To generate ground‐truth differentially abundant cell types, we defined the effect vector $\eta_{1,B}=\tau i\eta_{0,B}$, where i is a p‐dimensional binary vector that indicates the cell types that are influenced by the condition (i.e., are differentially abundant), and τ=(−0.5,−0.3,0.3,0.5,1) controls the relative effect size.

Using this model, we considered three differential abundance scenarios: (i) Estimation when the effect is on a rare cell type with (pDC), (ii) effect estimation on an abundant cell type (T4), and (iii) effects on both cell types (pDC and T4). Two different sample sizes (n=100 and n=1000) were considered for each case. For each of the resulting 30 scenarios, we generated five datasets with n/2 control samples ($K=K_{B}$, $\eta =\eta_{0,B}$) and n/2 case samples ($K=K_{B}$, $\eta =\eta_{0,B}+\eta_{1,B}$). To simulate these semi‐synthetic data sets, we used the adaptive rejection sampler from the *genscore* R package [34]. For completeness, we also considered an identical simulation scenario without correlated features with K=0. We show and discuss the results of this baseline scenario in Appendix D.

To illustrate the performance of cosmoDA for higher‐dimensional datasets, we conducted another set of simulations with p=99 features. We constructed the corresponding interaction matrix as a block‐diagonal matrix, using the original $K_{B}$ matrix in each of the nine blocks (see Figure F4a). Likewise, we stacked the scenario‐specific location vectors $\eta_{0,B}$ and $\eta_{1,B}$ nine times to obtain the high‐dimensional location vectors (Figure F4b).

We compared the ability of four different DA testing methods to recover differentially abundant features at an expected FDR level of α=0.05:

- DA testing with cosmoDA (a=b=0). We used $n_{\lambda }=20$ values between $10^{-3}$ and 2 for $\lambda_{1}$ and 5‐fold cross validation with the 1SE rule for model selection. All other parameters were set to default values described in Section 2.2.3.
- ANCOM‐BC [26] with default parameters as an example for a common DA testing method without feature interactions. Since ANCOM‐BC assumes count data instead of relative abundances, we scaled the simulated data by the median sequencing depth over all samples in the original dataset and rounded to the nearest integer to obtain comparable counts.
- A Dirichlet regression model and subsequent significance test on the regression coefficients, as implemented by [47]. This model serves as a simple baseline that does not respect feature interactions.
- CompDA [29], a recent DA testing method for compositional data, respecting feature interactions via conditional dependency modeling.

**FIGURE 4:**
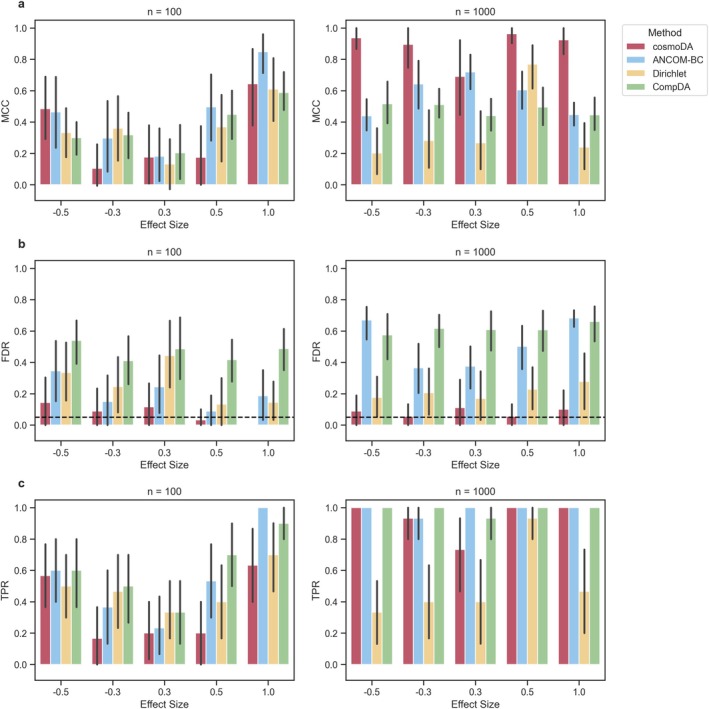
**Performance comparison for recovering differentially abundant features across different scenarios,** p=11. (a) Matthews' correlation coefficient, (b) False discovery rate. The dashed line shows the nominal FDR for all methods, and (c) True positive rate (power).

Figure 4 summarizes the results for the simulation scenario with the original number of features (p=11). Here, cosmoDA showed the overall best ability to recover the true effects (Matthews' correlation coefficient, Figure 4a), especially when the sample size was larger and for the more abundant cell type T4 (see Figure F5). Importantly, cosmoDA showed the lowest FDR value in all scenarios (Figure 4b). Although cosmoDA was not able to control the FDR at the expected level of 0.05 in every scenario, the methods without consideration of interactions (ANCOM‐BC and Dirichlet) detected more false positive features, with FDR levels averaging between 0.2 and 0.7 in most scenarios. Surprisingly, CompDA did not achieve lower FDR values than ANCOM‐BC and performed worse than Dirichlet regression in all cases. We observed slightly elevated FDR levels of cosmoDA in cases where the DA cell types were not detected, resulting in FDR values of 1 where one feature was falsely discovered (see Figure F6). While Dirichlet regression and ANCOM‐BC struggled with FDR control in all scenarios (see Figure F6), CompDA produced much higher FDR values for the abundant cell type (T4). For smaller sample sizes (n=100) and small effects, cosmoDA was not able to consistently detect the differentially abundant features, resulting in lower power for these scenarios. With increasing sample size, the power of cosmoDA was on par with the other methods (Figures 4c, F7).

**FIGURE 5:**
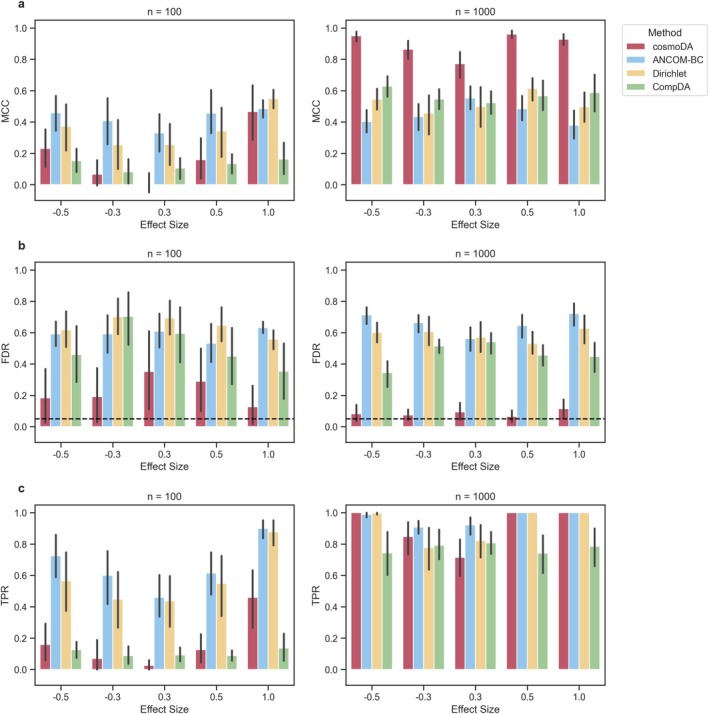
**Performance comparison for recovering differentially abundant features across different scenarios,** p=99. (a) Matthews' correlation coefficient, (b) False discovery rate. The dashed line shows the nominal FDR for all methods, and (c) True positive rate (power).

In the large‐dimensional case (p=99), the performance of all methods decreased in the small sample size scenario, while Matthews' correlation coefficient was similar to the case of p=11 for larger sample sizes (Figures 5a, F11). Again, cosmoDA always showed the lowest FDR, albeit with slightly elevated levels for n=1000, and mean FDR levels between 0.1 and 0.4 for n=100 (Figure 5b). The FDR levels for cosmoDA did not show a trend across feature rarity and effect size, while the other methods were not able to produce average FDR levels below 0.5 for effects on rare pDC cells (Figure F12). In terms of power, only ANCOM‐BC and Dirichlet regression were able to correctly detect some differentially abundant features for n=100, while all methods showed good power for larger sample sizes (Figure 5c). Breaking these results down by cell type revealed a good power of ANCOM‐BC and Dirichlet regression for abundant features, while effects on rare features could not be reliably detected by any method (Figure F13). The unsuitability of cosmoDA and CompDA for the case of p=99,n=100 is not surprising, as both models need to estimate pairwise feature interactions in the high‐dimensional regime.

## 4 Applications to Single‐Cell and Microbiome Data

To showcase the DA testing capabilities of cosmoDA on real data, we considered two compositional datasets: PBMC abundances derived from scRNA‐seq data of SLE patients (as used in the semi‐synthetic benchmarks [45]) and infant gut microbiome data from 16S rRNA sequencing [48]. Apart from comparing the empirical results with other state‐of‐the‐art methods, we also evaluated the impact of power transformations (a=b=ϕ≠0) on the downstream DA results.

### 4.1 DA Analysis of Cell Type Compositions in Patients With Systemic Lupus Erythematosus

We used the scRNA‐Seq‐derived PBMC data from Reference [45] (n=352,p=11, see Section 3.2) to estimate differences in cell type composition between subjects with systemic lupus erythematosus (SLE) (n = 208) and healthy controls (n = 144).

To tune the parameters in cosmoDA, we considered the power values ϕ=(0.01,0.02,0.03,…0.99), as well as the log‐log model (ϕ=0) for comparison. We set the range of $\lambda_{1}$ values between 1.5 and $10^{-7}$ to ensure full coverage of the range of supports of K for every value of ϕ. As before, we used NK cells as the reference cell type for cosmoDA and selected the regularization strength via 5‐fold cross‐validation with the 1SE rule. We used ANCOM‐BC, Dirichlet regression, and CompDA for comparison.

**FIGURE 6:**
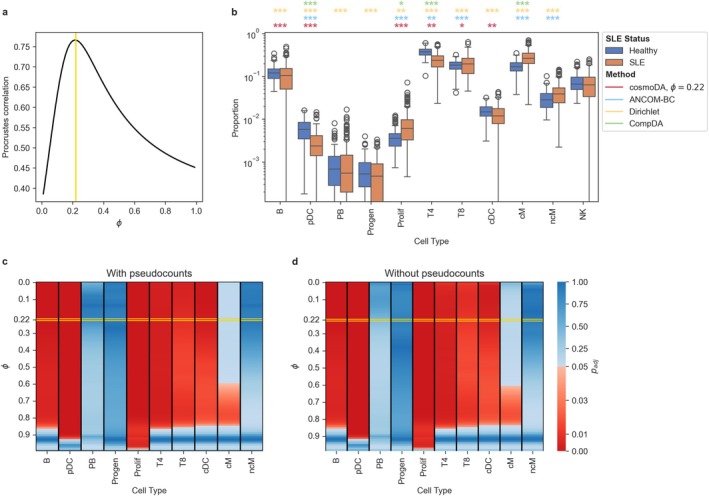
**Differential abundance testing with cosmoDA on the lupus dataset.** (a) Procrustes correlation between power transformation and ALR transformation with zero replacement. The yellow line ($\phi^{*}=0.22$) indicates the maximal Procrustes correlation. (b) Boxplot of relative abundance data without zero replacement. The colored stars indicate the significance level for each method (*: $p_{adj}<0.05$; **: $p_{adj}<0.01$; ***: $p_{adj}<0.001$). Differential abundance results on NK cells (reference in cosmoDA) are omitted. (c) Adjusted *p*‐values for testing differential abundance with cosmoDA on zero‐replaced data with different power transformations. Red entries denote differential abundance at a level of α=0.05, blue entries denote no differential abundance. The yellow box highlights the adjusted *p*‐values for $\phi^{*}$ determined in a. (d) Same as c, but using the raw data without zero replacement.

We first investigated the influence of our power value tuning schemes for DA analysis. The Procrustes correlation analysis showed that the ALR‐transformed PBMC data (with zeros replaced by a pseudocount of 0.5) and the power‐transformed data had the highest alignment for a power of $\phi^{*}=0.22$ (see Figure 6a). To investigate the impact of zero replacement and the power transform on differential abundance, we also compared the DA testing results of cosmoDA for all values of ϕ with and without zero entries (Figure 6c,d). Due to the low number of zeroes (4.5%), the impact of zero imputation was negligible for this dataset, making the adjusted *p*‐values with and without zero imputation almost identical. Below a value of 0.8, the exponent of the power transformation only impacted the differential abundance of CD14+ classical monocytes (cM). For higher exponents, almost all cell types showed no differential abundance. Comparing the results of cosmoDA with ANCOM‐BC, Dirichlet regression, and CompDA (all implemented as described in Section 3.2) showed that all methods selected different sets of differentially abundant cell types (Figure 6b). CompDA produced the most conservative results, only finding four DA cell types at an FDR level of 0.05. On the other hand, Dirichlet regression found all cell types to be differentially abundant. Interestingly, cosmoDA was the only method that did *not* select classical monocytes as differentially abundant (Figure 6b). The latter finding is in agreement with a control experiment performed by [45] that found absolute monocyte abundances to be not differentially abundant in SLE patients.

### 4.2 DA Analysis in Microbiome Data

To showcase the suitability of cosmoDA for microbial 16S rRNA sequencing, we used gut microbiome data from infants in Malawi and the United States [48]. We followed the pre‐processing in the original ANCOM‐BC study [26] and aggregated the data to the Phylum level. We selected all samples from subjects aged less than two years old in Malawi and the United States. Following [26], we next discarded all phyla where more than 90% of samples contained zero entries, resulting in n=97 samples of p=13 phyla. We selected Bacteroidetes as the reference phylum and applied cosmoDA with ϕ=(0.01,0.02,0.03,…0.99), and the log‐log model (ϕ=0) to the relative abundances with and without zero replacement. The range of values for $\lambda_{1}$ was set to $\left[10^{-12},\;1.5\right]$, and we used 5‐fold cross‐validation with the 1SE rule to select $\lambda_{1}$ for each value of ϕ.

**FIGURE 7:**
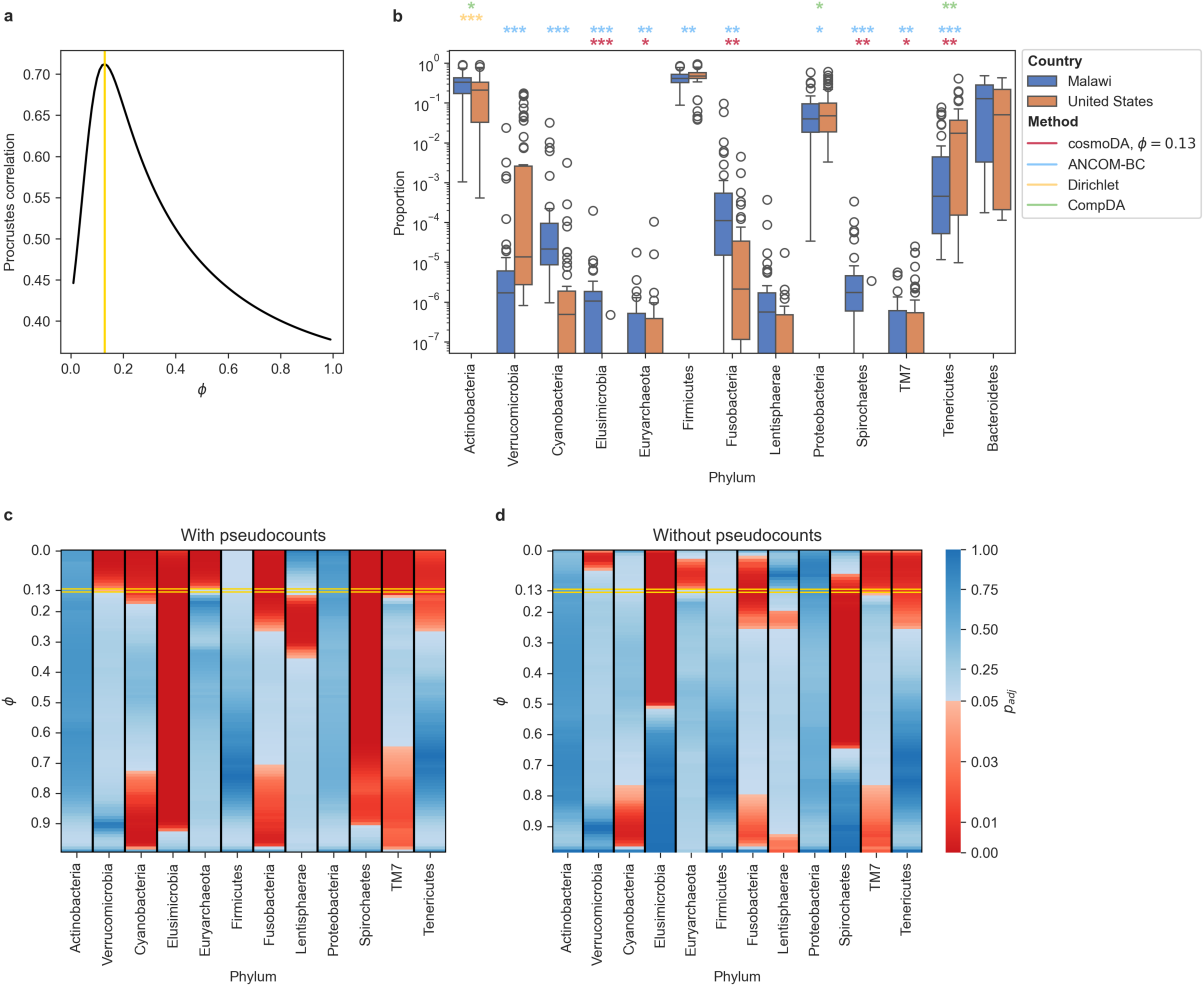
**Differential abundance testing (US vs. Malawi) with cosmoDA on infants (age** <2 **years) in the human gut dataset.** (a) Procrustes correlation between power transformation and ALR transformation with zero replacement. The yellow line ($\phi^{*}=0.13$) indicates the maximal Procrustes correlation. (b) Boxplot of relative abundance data without zero replacement. The colored stars indicate the significance level for each method (*: $p_{adj}<0.05$; **: $p_{adj}<0.01$; ***: $p_{adj}<0.001$). Differential abundance results on Bacteroidetes (reference in cosmoDA) are omitted. (c) Adjusted *p*‐values for testing differential abundance with cosmoDA on zero‐replaced data with different power transformations. Red entries denote differential abundance at a level of α=0.05, blue entries denote no differential abundance. The yellow box highlights the adjusted *p*‐values for $\phi^{*}$ determined in a. (d) Same as c, but using the raw data without zero replacement.

For this dataset, our power selection scheme identified $\phi^{*}=0.13$ to result in the best Procrustes alignment (see Figure 7a). The larger proportion of zero entries in this dataset (28.6%) caused more differences in downstream DA testing results, both on the original and zero‐imputed data (Figure 7c,d). While the DA pattern of taxa with no zero entries (Firmicutes, Actinobacteria, Tenericutes, and Proteobacteria) was not impacted by zero imputation, the phyla with at least 20% zero entries (Cyanobacteria, Elusimicrobia, Euryarchaeota, Lentispherae, Spirochaetes, and TM7) were deemed differentially abundant at a smaller power value. Similar to the analysis of the scRNA‐seq data, the four DA methods produced different sets of DA taxa at an FDR level of 0.05 (Figure 7b). Dirichlet regression and CompDA seemed to be only sensitive to taxa with high average abundance, while ANCOM‐BC and cosmoDA were also able to detect differential abundance in rare phyla. The set of DA taxa discovered by cosmoDA at $\phi^{*}=0.13$ on the data with zero entries was smaller than the set discovered on the same dataset by ANCOM‐BC. Nevertheless, cosmoDA found multiple phyla that are associated with rural lifestyles (Elusimicrobia, Euryarchaeota, Spirochaetes) to be increased in infants from Malawi [49, 50]. Notably, the ANCOM‐BC algorithm involves the replacement of zeros by a small pseudocount [26]. Indeed, the set of DA phyla discovered by cosmoDA on the zero‐replaced data with the exponent $\phi^{*}=0.13$ (Figure 7c) almost perfectly matched the DA phyla found by ANCOM‐BC (except Firmicutes and Proteobacteria). Overall, this confirms that the replacement of zero entries in microbial abundance data has a significant impact on differential abundance.

## 5 Conclusion

Tissues and bacterial communities are complex biological environments, governed by interactions between individual cell types or microbial taxa. The prevailing high‐throughput sequencing (HTS) data sets probing these complex mixtures are often compositional in nature. Statistical generative modeling, as well as differential abundance testing schemes for such compositional datasets, can therefore suffer from inaccuracies if interactions between cells or microbes are not considered in the analysis. Extending the class of a‐b power interaction models [24] by a linear effect on the location vector, our new method cosmoDA allows accurate modeling of HTS data with pairwise feature interactions in the presence of covariate information. The covariate formulation in cosmoDA also seamlessly integrates into the generalized score matching optimization framework [25, 35, 36], facilitating fast and accurate parameter inference. $L_{1}$ regularization on the interaction matrix further avoids model complexity explosion and allows to select parsimonious interaction patterns. Compared to the a‐b power interaction model without covariates from Reference [24], the addition of a covariate did not reduce its ability to detect significant feature interactions in our synthetic data simulations. Both the covariate‐less and covariate‐extended a‐b power interaction models outperformed other established procedures for identifying sparse interactions in compositional HTS data when the sample size was sufficiently large.

In the presence of a binary condition, testing for the significance of the covariate‐related parameters in the location vector acts as a form of differential abundance testing. Here, the parallel estimation of feature interactions helps to avoid false positive detections, which are only indirectly related to the condition. In our realistic simulation experiments, cosmoDA was the only method to approximately control the false discovery rate in the presence of feature interactions, while no other tested method could distinguish between direct and indirect compositional changes. cosmoDA showed reduced power when the sample size was small, but was on par with methods like ANCOM‐BC [26] for larger numbers of observations. One exception where cosmoDA was not able to adequately control the FDR was for misspecified models with more features than samples. We further demonstrated the ability of cosmoDA to find biologically meaningful differential abundances on two experimental datasets from human single‐cell RNA sequencing and microbiome 16S rRNA sequencing.

The use of power transformations instead of the logarithm in a‐b power interaction models allows to keep zero measurements in the data as is, avoiding distortions caused by imputation of these values. Through a small adjustment in the score matching optimizer, we were able to approximate the log‐transformation for exponents approaching zero. Applying cosmoDA to real‐world single‐cell and microbiome datasets, we discovered that zero replacement and the exponent of the power transformation had a considerable impact on downstream DA results in data with excess zeros [8]. We further demonstrated that selecting an exponent for the power transformation that approximates the data geometry after an ALR transformation generally produces sensible differential abundance results.

While cosmoDA successfully tackles multiple challenges in generative modeling and differential abundance testing, it also has some limitations. Currently, cosmoDA can only accommodate a single binary or continuous covariate. Extending the linear model formulation would allow to model more complex scenarios and adjusting for confounders in DA testing. For this, the score matching estimator would also have to be extended to multiple covariates. The implementation of such a flexible model could be simplified by using automatic differentiation for determining the elements of Γ and g [51]. In addition, we believe that approximation of the logarithm for small exponents can be solved more elegantly by changing the general definition of a‐b power interaction models to utilize a true Box–Cox transformation rather than using our proposed adjustments in the score matching optimizer. Estimation of our model also relies on selecting a good reference, which is profiled out in the model formulation. Looping over multiple references and averaging the results, as described by [24], could avoid this dependency at the cost of computational efficiency.

While we empirically showed the feasibility of cosmoDA, we did not provide extensive formal guarantees for goodness of fit and convergence. Formally evaluating and extending our brief discussion of how the convergence criteria for a‐b power interaction models provided by [24] are applicable to our model (Section 2.2.2) would give more justification to our approach. Similarly, a thorough goodness‐of‐fit comparison of our model and other approaches for modeling compositional high‐throughput sequencing data (e.g., [3, 22, 32]) on a wide variety of real datasets could help characterize the strengths and weaknesses of different parametric and nonparametric approaches further. While such an evaluation is beyond the scope of this work, possible metrics for such a comparison could include the Fisher divergence, Kernelized Stein discrepancy [52], or simulation‐based predictive comparisons.

Furthermore, cosmoDA requires any count data to be transformed to relative abundance data and treated as quasi‐continuous. Due to this choice, inference of effects on very rare features can become imprecise due to the discrete lattice structure of count data [53]. In our approach, this effect can only be mitigated by conducting experiments with higher sequencing depth.

Overall, we believe that cosmoDA, with its abilities to include feature interactions and seamless handling of excess zeros, represents a valuable addition to the growing family of differential abundance testing methods. A Python implementation of cosmoDA and the power interaction model without covariates is available at https://github.com/bio‐datascience/cosmoDA.

## Author Contributions

J.O. developed the idea of cosmoDA with help from H.L. and C.L.M. J.O. implemented the method, performed and evaluated all simulations, and conducted the data applications. J.O. wrote the manuscript with assistance from C.L.M. All authors read and approved the final manuscript.

## Funding

This work was supported by the Helmholtz‐Gemeinschaft (Grant No. ZT‐I‐PF‐5‐138) and National Institutes of Health (Grant No. R01GM123056).

## Conflicts of Interest

The authors declare no conflicts of interest.

## Supporting information




**Data S1**: Online Appendix.

## Data Availability

All datasets used in this article are publicly available. The SLE scRNA‐seq data were downloaded from the Human Cell Atlas platform (GSE174188). The gut microbiome data is stored at MG‐RAST https://www.mg‐rast.org/index.html under search string “mgp401”, code for data preparation was adapted from https://github.com/FrederickHuangLin/ANCOM‐BC. Code for reproducing the analyses in this article is available under https://github.com/bio‐datascience/cosmoDA, intermediate data can be found at https://zenodo.org/records/13911623.
