# Supplementary material for: Score Matching for Differential Abundance Testing of Compositional High‐Throughput Sequencing Data
Source: Stat Med. 2026 Apr 7;45(8-9):e70534. doi: 10.1002/sim.70534 (PMC13055433; doi:10.1002/sim.70534)
Supplement: Supplementary file 1 — Data S1: Online Appendix. [file SIM-45-0-s001.pdf]

Appendix

A | CONNECTION BETWEEN A-B POWER INTERACTION MODELS AND PPI MODELS

The class of polynomially tilted pairwise interaction (PPI) models, introduced by[23], is another class of flexible distributions with feature interactions on the simplex. This class includes distributions of the form

$$p_{\boldsymbol{A}^*,\boldsymbol{\nu}}(\boldsymbol{x}) \propto \left(\prod_{i=1}^{p} x_i^{\nu_i}\right) \exp(\boldsymbol{x}^T \boldsymbol{A}^* \boldsymbol{x})$$

with $\boldsymbol{\nu} \succ -1 \in \mathbb{R}^p$, and $\boldsymbol{A}^* \in \mathbb{R}^{p\times p}$ symmetric with $\boldsymbol{A}^*\mathbf{1}_p = 0$. Through $\left(\prod_{i=1}^{p} x_i^{\nu_i}\right) = \exp(\boldsymbol{\nu}^T \log(\boldsymbol{x}))$, it is easy to see that this class of distributions represents a special case of a-b power interaction models (Eq. 2) with $a = 1$ and $b = 0$. Profiling out the last coordinate, i.e. $x_p = 1 - \sum_{i=1}^{p-1} x_i$, leads to an alternative formulation[24], with parameters $\boldsymbol{\nu} \succ -1 \in \mathbb{R}^p$, $\boldsymbol{A}_L \in \mathbb{R}^{(p-1)\times(p-1)}$, and $\boldsymbol{c}_L \in \mathbb{R}^{(p-1)}$:

$$p_{\boldsymbol{A}_L,\boldsymbol{c}_L,\boldsymbol{\nu}}(\boldsymbol{x}) \propto \left(\prod_{i=1}^{p} x_i^{\nu_i}\right) \exp(\boldsymbol{x}^T \boldsymbol{A}_L \boldsymbol{x} + \boldsymbol{c}_L^T \boldsymbol{x}).$$

In particular, the transition between the two forms can be achieved by splitting off the last row and column of $\boldsymbol{A}^* = \begin{pmatrix} \boldsymbol{A}_L^* & \boldsymbol{A}_p^* \\ \boldsymbol{A}_p^{*T} & \boldsymbol{A}_{pp}^* \end{pmatrix}$. Then, $\boldsymbol{A}_{Li,j} = \boldsymbol{A}^*{}_{i,j} - 2\boldsymbol{A}^*_{p\,i} + \boldsymbol{A}^*_{pp}$ and $\boldsymbol{c}_{Li} = 2(\boldsymbol{A}^*_{p\,i} - \boldsymbol{A}^*_{pp})$. Since $\boldsymbol{A}^*$ has one additional parameter, assume $\boldsymbol{A}^*_{pp} = 0$ for the reverse transformation. Then, $\boldsymbol{A}^*_{p\,i} = \frac{1}{2}\boldsymbol{c}_{Li}$, and $\boldsymbol{A}^*_{Li,j} = \boldsymbol{A}_{Li,j} + \boldsymbol{c}_{Li}$.

Applying the equivalent transformations to an a-b power interaction model with $a = 1$ can help with parameter interpretation, as the matrix $\boldsymbol{A}_L$ usually has full rank.

B | DERIVATION OF THE PARAMETERS IN THE QUADRATIC FORM OF THE SCORE MATCHING OPTIMIZER

This section details the derivation of the parameters $\boldsymbol{\Gamma}$ and $\boldsymbol{g}$ in the quadratic formulation of the score matching loss (Eq. 8) and explains their block structure shown in Eq. 11. The elements of $\boldsymbol{g}$ can be directly derived from the second derivative of $\log p(\boldsymbol{x})$ (Eq. 10):

$$\begin{aligned}
\boldsymbol{g}_{\mathbf{K},j} &\equiv \frac{1}{n}\sum_{i=1}^{n}\left[\partial_j \tilde{h}_j\left(\boldsymbol{X}^{(i)}\right) X_j^{(i)^{a-1}} + (a-1)\tilde{h}_j\left(\boldsymbol{X}^{(i)}\right) X_j^{(i)^{a-2}}\right] \boldsymbol{X}^{(i)^{a}} \\
&\quad + a\tilde{h}_j\left(\boldsymbol{X}^{(i)}\right) X_j^{(i)^{2a-2}} \boldsymbol{e}_{j,p} - a\tilde{h}_j\left(\boldsymbol{X}^{(i)}\right) X_j^{(i)^{a-1}} X_p^{(i)^{a-1}} \boldsymbol{e}_{p,p}, \\
\boldsymbol{g}_{\mathbf{K},p} &\equiv \frac{1}{n}\sum_{i=1}^{n}\sum_{k=1}^{p-1}\left[-\partial_k \tilde{h}_k\left(\boldsymbol{X}^{(i)}\right) X_p^{(i)^{a-1}} + (a-1)\tilde{h}_k\left(\boldsymbol{X}^{(i)}\right) X_p^{(i)^{a-2}}\right] \boldsymbol{X}^{(i)^{a}} \\
&\quad + a\tilde{h}_k\left(\boldsymbol{X}^{(i)}\right) X_p^{(i)^{2a-2}} \boldsymbol{e}_{p,p} - a\tilde{h}_k\left(\boldsymbol{X}^{(i)}\right) X_k^{(i)^{a-1}} X_p^{(i)^{a-1}} \boldsymbol{e}_{k,p}, \\
\boldsymbol{g}_{\boldsymbol{\eta}_0,j} &\equiv \frac{1}{n}\sum_{i=1}^{n} -\partial_j \tilde{h}_j\left(\boldsymbol{X}^{(i)}\right) X_j^{(i)^{b-1}} - (b-1)\tilde{h}_j\left(\boldsymbol{X}^{(i)}\right) X_j^{(i)^{b-2}} = \frac{1}{n}\sum_{i=1}^{n} \boldsymbol{g}^{(i)}_{\boldsymbol{\eta}_1,j}, \\
\boldsymbol{g}_{\boldsymbol{\eta}_0,p} &\equiv \frac{1}{n}\sum_{i=1}^{n}\sum_{k=1}^{p-1} \partial_k \tilde{h}_k\left(\boldsymbol{X}^{(i)}\right) X_p^{(i)^{b-1}} - (b-1)\tilde{h}_k\left(\boldsymbol{X}^{(i)}\right) X_p^{(i)^{b-2}} = \frac{1}{n}\sum_{i=1}^{n} \boldsymbol{g}^{(i)}_{\boldsymbol{\eta}_1,p}, \\
\boldsymbol{g}_{\boldsymbol{\eta}_1,j} &\equiv \frac{1}{n}\sum_{i=1}^{n} -\partial_j \tilde{h}_j\left(\boldsymbol{X}^{(i)}\right) yX_j^{(i)^{b-1}} - (b-1)\tilde{h}_j\left(\boldsymbol{X}^{(i)}\right) yX_j^{(i)^{b-2}} = \frac{1}{n}\sum_{i=1}^{n} y\boldsymbol{g}^{(i)}_{\boldsymbol{\eta}_1,j}, \\
\boldsymbol{g}_{\boldsymbol{\eta}_1,p} &\equiv \frac{1}{n}\sum_{i=1}^{n}\sum_{k=1}^{p-1} \partial_k \tilde{h}_k\left(\boldsymbol{X}^{(i)}\right) yX_p^{(i)^{b-1}} - (b-1)\tilde{h}_k\left(\boldsymbol{X}^{(i)}\right) yX_p^{(i)^{b-2}} = \frac{1}{n}\sum_{i=1}^{n} y\boldsymbol{g}^{(i)}_{\boldsymbol{\eta}_1,p}.
\end{aligned}$$

Further, the elements of $\boldsymbol{\Gamma}$ follow from the first derivative of $\log p(\boldsymbol{x})$ (Eq. 9) and have the same structure as in[1]:

$$\mathbf{\Gamma_K} \equiv \begin{bmatrix} \mathbf{\Gamma}_{\mathbf{K},1} & \mathbf{0} & \cdots & \mathbf{0} & \mathbf{\Gamma}_{\mathbf{K},(1,p)} \\ \mathbf{0} & \mathbf{\Gamma}_{\mathbf{K},2} & \cdots & \mathbf{0} & \mathbf{\Gamma}_{\mathbf{K},(2,p)} \\ \vdots & \vdots & \ddots & \vdots & \vdots \\ \mathbf{0} & \mathbf{0} & \cdots & \mathbf{\Gamma}_{\mathbf{K},p-1} & \mathbf{\Gamma}_{\mathbf{K},(p-1,p)} \\ \mathbf{\Gamma}_{\mathbf{K},(1,p)}^\top & \mathbf{\Gamma}_{\mathbf{K},(2,p)}^\top & \cdots & \mathbf{\Gamma}_{\mathbf{K},(p-1,p)}^\top & \mathbf{\Gamma}_{\mathbf{K},p} \end{bmatrix} \in \mathbb{R}^{p^2\times p^2},$$

with each block of size $p \times p$, and

$$\mathbf{\Gamma}_{\mathbf{K},\boldsymbol{\eta}_i} \equiv \begin{bmatrix} \boldsymbol{\gamma}_{\mathbf{K},\boldsymbol{\eta}_i,1} & \mathbf{0} & \cdots & \mathbf{0} & \boldsymbol{\gamma}_{\mathbf{K},\boldsymbol{\eta}_i,(1,p)} \\ \mathbf{0} & \boldsymbol{\gamma}_{\mathbf{K},\boldsymbol{\eta}_i,2} & \cdots & \mathbf{0} & \boldsymbol{\gamma}_{\mathbf{K},\boldsymbol{\eta}_i,(2,p)} \\ \vdots & \vdots & \ddots & \vdots & \vdots \\ \mathbf{0} & \mathbf{0} & \cdots & \boldsymbol{\gamma}_{\mathbf{K},\boldsymbol{\eta}_i,p-1} & \boldsymbol{\gamma}_{\mathbf{K},\boldsymbol{\eta}_i,(p-1,p)} \\ \boldsymbol{\gamma}_{\mathbf{K},\boldsymbol{\eta}_i,(p,1)} & \boldsymbol{\gamma}_{\mathbf{K},\boldsymbol{\eta}_i,(p,2)} & \cdots & \boldsymbol{\gamma}_{\mathbf{K},\boldsymbol{\eta}_i,(p,p-1)} & \boldsymbol{\gamma}_{\mathbf{K},\boldsymbol{\eta}_i,p} \end{bmatrix} \in \mathbb{R}^{p^2\times p} \textit{ for } i \in \{1,2\},$$

with each block a vector of size p, and

$$\mathbf{\Gamma}_{\boldsymbol{\eta}_i} \equiv \begin{bmatrix} \gamma_{\boldsymbol{\eta}_i,1} & 0 & \cdots & 0 & \gamma_{\boldsymbol{\eta}_i,(1,p)} \\ 0 & \gamma_{\boldsymbol{\eta}_i,2} & \cdots & 0 & \gamma_{\boldsymbol{\eta}_i,(2,p)} \\ \vdots & \vdots & \ddots & \vdots & \vdots \\ 0 & 0 & \cdots & \gamma_{\boldsymbol{\eta}_i,p-1} & \gamma_{\boldsymbol{\eta}_i,(p-1,p)} \\ \gamma_{\boldsymbol{\eta}_i,(1,p)} & \gamma_{\boldsymbol{\eta}_i,(2,p)} & \cdots & \gamma_{\boldsymbol{\eta}_i,(p-1,p)} & \gamma_{\boldsymbol{\eta}_i,p} \end{bmatrix} \in \mathbb{R}^{p\times p} \textit{ for } i \in \{1,2\},$$

, and

$$\mathbf{\Gamma}_{\boldsymbol{\eta}_0,\boldsymbol{\eta}_1} \equiv \begin{bmatrix} \gamma_{\boldsymbol{\eta}_0,\boldsymbol{\eta}_1,1} & 0 & \cdots & 0 & \gamma_{\boldsymbol{\eta}_0,\boldsymbol{\eta}_1,(1,p)} \\ 0 & \gamma_{\boldsymbol{\eta}_0,\boldsymbol{\eta}_1,2} & \cdots & 0 & \gamma_{\boldsymbol{\eta}_0,\boldsymbol{\eta}_1,(2,p)} \\ \vdots & \vdots & \ddots & \vdots & \vdots \\ 0 & 0 & \cdots & \gamma_{\boldsymbol{\eta}_0,\boldsymbol{\eta}_1,p-1} & \gamma_{\boldsymbol{\eta}_0,\boldsymbol{\eta}_1,(p-1,p)} \\ \gamma_{\boldsymbol{\eta}_0,\boldsymbol{\eta}_1,(1,p)} & \gamma_{\boldsymbol{\eta}_0,\boldsymbol{\eta}_1,(2,p)} & \cdots & \gamma_{\boldsymbol{\eta}_0,\boldsymbol{\eta}_1,(p-1,p)} & \gamma_{\boldsymbol{\eta}_0,\boldsymbol{\eta}_1,p} \end{bmatrix} \in \mathbb{R}^{p\times p}.$$

These blocks have the following specific forms. For $j = 1, \ldots, p-1$,

$$\boldsymbol{\Gamma}_j \equiv \begin{bmatrix} \boldsymbol{\Gamma}_{\mathbf{K},j} & \gamma_{\mathbf{K},\boldsymbol{\eta}_0,j} & \gamma_{\mathbf{K},\boldsymbol{\eta}_1,j} \\ \gamma_{\mathbf{K},\boldsymbol{\eta}_0,j}^\top & \gamma_{\boldsymbol{\eta}_0,j} & \gamma_{\boldsymbol{\eta}_0,\boldsymbol{\eta}_1,j} \\ \gamma_{\mathbf{K},\boldsymbol{\eta}_1,j}^\top & \gamma_{\boldsymbol{\eta}_0,\boldsymbol{\eta}_1,j}^\top & \gamma_{\boldsymbol{\eta}_1,j} \end{bmatrix}$$
$$\equiv \frac{1}{n}\sum_{i=1}^{n} \tilde{h}_j\left(\boldsymbol{X}^{(i)}\right) \begin{bmatrix} X_j^{(i)a-1}\boldsymbol{X}^{(i)a} \\ -X_j^{(i)b-1} \\ -yX_j^{(i)b-1} \end{bmatrix} \begin{bmatrix} X_j^{(i)a-1}\boldsymbol{X}^{(i)a} \\ -X_j^{(i)b-1} \\ -yX_j^{(i)b-1} \end{bmatrix}^\top,$$

$$\boldsymbol{\Gamma}_p \equiv \begin{bmatrix} \boldsymbol{\Gamma}_{\mathbf{K},p} & \gamma_{\mathbf{K},\boldsymbol{\eta}_0,p} & \gamma_{\mathbf{K},\boldsymbol{\eta}_1,p} \\ \gamma_{\mathbf{K},\boldsymbol{\eta}_0,p}^\top & \gamma_{\boldsymbol{\eta}_0,p} & \gamma_{\boldsymbol{\eta}_0,\boldsymbol{\eta}_1,p} \\ \gamma_{\mathbf{K},\boldsymbol{\eta}_1,p}^\top & \gamma_{\boldsymbol{\eta}_0,\boldsymbol{\eta}_1,p}^\top & \gamma_{\boldsymbol{\eta}_1,p} \end{bmatrix}$$
$$\equiv \frac{1}{n}\sum_{i=1}^{n}\sum_{k=1}^{p-1} \tilde{h}_k\left(\boldsymbol{X}^{(i)}\right) \begin{bmatrix} X_p^{(i)a-1}\boldsymbol{X}^{(i)a} \\ -X_p^{(i)b-1} \\ -yX_p^{(i)b-1} \end{bmatrix} \begin{bmatrix} X_p^{(i)a-1}\boldsymbol{X}^{(i)a} \\ -X_p^{(i)b-1} \\ -yX_p^{(i)b-1} \end{bmatrix}^\top,$$

$$\boldsymbol{\Gamma}_{(j,p)} \equiv \begin{bmatrix} \boldsymbol{\Gamma}_{\mathbf{K},(j,p)} & \gamma_{\mathbf{K},\boldsymbol{\eta}_0,(j,p)} & \gamma_{\mathbf{K},\boldsymbol{\eta}_1,(j,p)} \\ \gamma_{\mathbf{K},\boldsymbol{\eta}_0,(p,j)} & \gamma_{\boldsymbol{\eta}_0,(j,p)} & \gamma_{\boldsymbol{\eta}_0,\boldsymbol{\eta}_1,(j,p)} \\ \gamma_{\mathbf{K},\boldsymbol{\eta}_1,(p,j)} & \gamma_{\boldsymbol{\eta}_0,\boldsymbol{\eta}_1,(p,j)} & \gamma_{\boldsymbol{\eta}_1,(j,p)} \end{bmatrix}$$
$$\equiv -\frac{1}{n}\sum_{i=1}^{n} \tilde{h}_j\left(\boldsymbol{X}^{(i)}\right) \begin{bmatrix} X_j^{(i)a-1}\boldsymbol{X}^{(i)a} \\ -X_j^{(i)b-1} \\ -yX_j^{(i)b-1} \end{bmatrix} \begin{bmatrix} X_p^{(i)a-1}\boldsymbol{X}^{(i)a} \\ -X_p^{(i)b-1} \\ -yX_p^{(i)b-1} \end{bmatrix}^\top.$$

C | SCALING SCORE MATCHING ELEMENTS TO APPROXIMATE BOX-COX TRANSFORMATIONS

As described in Section 2.4.2, the power transformation used for a-b power interaction models (Eqs. 4 and 2) bears striking resemblance to the Box-Cox transformation $\frac{1}{\phi}(x^\phi - 1)$. Both transformations are not equivalent though due to the subtraction of 1 in the Box-Cox transformation. This difference causes the a-b power interaction transformation to lose one key property of the Box-Cox transformation - its asymptotic approximation of the logarithm as ϕ approaches 0.

Looking at the density of the covariate-extended a-b power interaction model makes this disparity clear:

$$p_{\boldsymbol{\eta},\mathbf{K}}(\boldsymbol{x}) \propto \exp\left(-\frac{1}{2a}\boldsymbol{x}^{a\top}\mathbf{K}\boldsymbol{x}^a + \frac{1}{b}(\eta_0 + \boldsymbol{y}\boldsymbol{\eta}_1)^\top \boldsymbol{x}^b\right)$$

For the terms concerning $\boldsymbol{\eta}_0$ and $\boldsymbol{\eta}_1$, the subtraction of 1 in the Box-Cox transformation is not dependent on x and can therefore be absorbed into the normalizing constant. For the interaction term, replacing $\boldsymbol{x}^a$ with the Box-Cox transformation in Eqs. 2 or 4 would introduce a scaling factor of order $1/a^2$ instead of $1/a$, leading to a discontinuity of the estimated $\boldsymbol{K}$ when approaching the log-log model, for which the convention $\frac{1}{2a} \equiv 1$ is used[1].

We counteract this effect by introducing scaling factors of $1/a$ and $1/a^2$ on the components of $\boldsymbol{\Gamma}$ and $\boldsymbol{g}$ (Eq. 11), based on the matrix multiplication $\boldsymbol{\theta}^\top\boldsymbol{\Gamma}(\mathbf{x})\boldsymbol{\theta}$ from Eq. 8. In particular, we scale $\boldsymbol{\Gamma}_{\boldsymbol{K}}$ by a factor of $\frac{1}{a^2}$ and $\boldsymbol{\Gamma}_{\boldsymbol{K},\boldsymbol{\eta}_0}$, $\boldsymbol{\Gamma}_{\boldsymbol{K},\boldsymbol{\eta}_1}$, and $\boldsymbol{g}_{\boldsymbol{K}}$ by a factor of $\frac{1}{a}$ each. This leads to a smooth transition in the estimation of $\boldsymbol{K}$ when $\phi \to 0$, and also holds for general a-b power interaction models without covariates[1].

We showcase the effectiveness of our scaling approach with an example on the scRNA-seq data of SLE patients and healthy controls[45]. For simplicity, we estimate the whole dataset through the covariate-less a-b power interaction model without differentiating between the two groups, use no regularization on the off-diagonal entries of $\boldsymbol{K}$, and always replace zeros with a value of 0.5. Without the scaling factor, the pattern of the estimated $\boldsymbol{K}$ approaches the log-solution ($\phi = 0$), but the scale of the entries is not the same (Figure F14, left column). On the other hand, the entries of $\boldsymbol{\eta}$ approach the log-solution also in magnitude (Figure F14, right column). For increasing values of ϕ, both the pattern and magnitude of $\boldsymbol{K}$ and $\boldsymbol{\eta}$ gradually diverge, as the power transformation gradually distorts the composition differently.

The median entry of the ratio $\boldsymbol{K}_{\phi=0}/\boldsymbol{K}_{\phi=\phi'}$ also does not approach 1 as $\phi' \to 0$ (Figure F15, bottom right). Looking at the components of $\boldsymbol{\Gamma}$ and $\boldsymbol{g}$, one can see that the median entry of the above ratio follows a log-linear trend for larger values of ϕ, but not for smaller exponents if the component is associated with $\boldsymbol{K}$ (Figure F15, other panels). The scaling factors introduced above correct this trend, such that the ratio is log-linear across the full spectrum of ϕ. This causes the estimated $\boldsymbol{K}$ to approach the solution for $\phi = 0$ in magnitude (Figure F15, bottom right) without impacting the estimated interaction pattern (Figure F14, middle column) or the estimation of $\boldsymbol{\eta}$ (Figure F14, right column).

When combining regularization and power transforms, the dependency between ϕ and the scale of entries in $\boldsymbol{K}$ will lead to different optimal regularization strengths for different exponents (Figure F16a). In fact, a larger exponent and therefore larger scale of $\boldsymbol{K}$ will require smaller values of λ_1 to cover the whole range between $\boldsymbol{K}$ with full support and a diagonal $\boldsymbol{K}$ (Figure F16b). Therefore, the range of values for λ_1 should always be adapted to the current data and power transform.

D | BASELINE DA SCENARIO: TESTING FOR DIFFERENTIAL ABUNDANCE IN THE ABSENCE OF CORRELATED FEATURES

To show the suitability of `cosmoDA` even in the case when no feature interactions are present, we considered the same setup as in 3.2 but without correlated features. To this end, we applied the a-b power model with $a = b = 0$ and $\lambda_1 = 2$ to the dataset from[45], resulting in ground truth parameters of $K_B = \mathbf{0}^{11\times 11}$, and $\boldsymbol{\eta}_{0,B}$ as shown in Figure F3. We used the same setup as before to select differentially abundant cell types and effect sizes and again chose $n = 100$ and $n = 1000$, simulating five replicates for each of the 30 scenarios as described in Section 3.2.

In this scenario, `cosmoDA` and CompDA showed similar overall performance as before, while the MCC of ANCOM-BC and Dirichlet regression significantly improved (Figures D1a, F8). This improvement was due to a reduction in falsely discovered effects by these methods (Figure D1a), which shows that the high FDR of ANCOM-BC and Dirichlet regression in the simulation scenario with correlated features were indeed caused by secondary effects due to feature interactions. In terms of power, all methods showed similar strength as before (Figures D1c, F10). Nevertheless, `cosmoDA` was the only model to consistently produce a FDR close to the nominal level, while CompDA was not able to avoid false discoveries if the effect was placed on the abundant cell type T4 (Figure F9). The superior performance of `cosmoDA` in this case is likely due to the fact that the data were simulated by an a-b power interaction model, deviating from the model assumptions of the other methods.

E | BENCHMARK: MISSPECIFIED MODEL AND EFFECTS ON RARE FEATURES

To show the applicability of `cosmoDA` to datasets that follow a different compositional distribution than the a-b power interaction model family, we conducted another simulated data benchmark. For this, we used the data simulator included in the scCODA package[4] to generate data under a Dirichlet-Multinomial distribution. We simulated $n = 200$ or $n = 500$ samples with $p = 10$ features and a baseline distribution vector of $\mu_0 = (0.14, 0.0005, 0.0005, 0.2, 0.009, 0.4, 0.05, 0.05, 0.05, 0.1) \cdot 20$ each. On half of the samples, we placed (additive) effects of $0.5, 0.75, 1, 1.5$, or 3 on the second feature. Considering 20 replicates for each combination of sample size and effect value, we simulated a total of 200 datasets with 2000 counts per sample. Since its baseline abundance is very low, the simulated datasets contained between 20% and 48% zero entries for the differentially abundant feature. This simultaneously allowed us to assess the performance of `cosmoDA` for rare features. We compared the same four methods as in the other benchmarks without replacing zero entries in the data. For ANCOM-BC[26], we used the generated counts as-is, while we transformed the data to relative abundances for `cosmoDA`, Dirichlet regression[47], and CompDA[29]. For every dataset, we also estimated a suitable exponent for the a-b power interaction model in `cosmoDA` by the procedure described in section 2.4.2.

Figure E1 shows that for lager effect sizes (≥ 1), `cosmoDA` performed similar to ANCOM-BC and CompDA and better than Dirichlet regression in terms of Matthews' correlation coefficient (Figure E1a). In addition, `cosmoDA` and ANCOM-BC were the only methods to appropriately control the false discovery rate in most scenarios, while CompDA and Dirichlet regression showed inflated FDR for larger effect sizes (Figure E1b). For small effects, `cosmoDA` detected the differentially abundant feature less often than ANCOM-BC and CompDA (Figure E1c). Still, `cosmoDA` showed overall similar performance than other state-of-the-art models in a misspecified data scenario. This is especially remarkable, as the Dirichlet distribution assumes no relationship between the features apart from the compositional correlation effect, eliminating the advantage of `cosmoDA` due to the estimation of pairwise feature interactions.

F SUPPLEMENTARY FIGURES

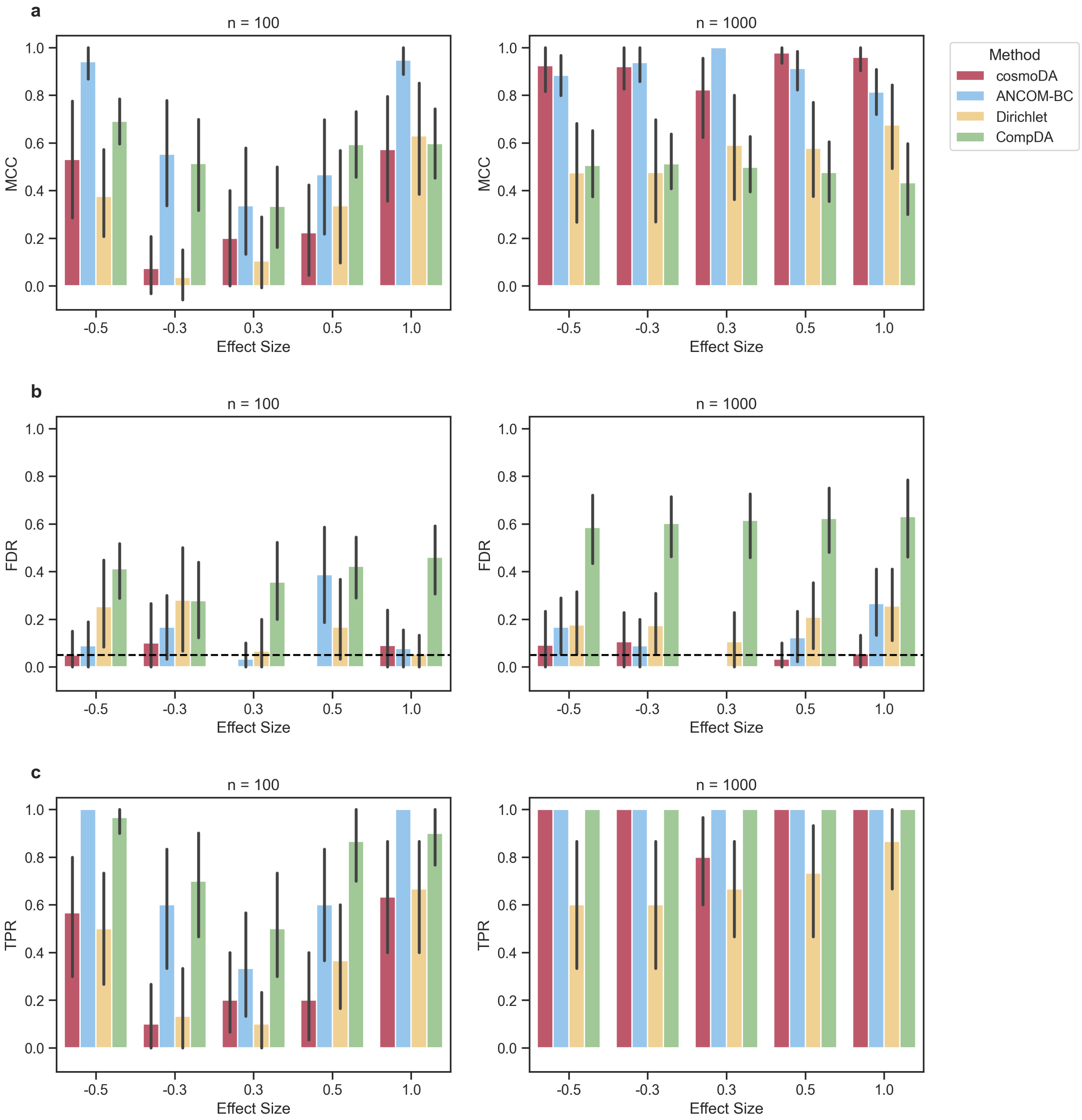

FIGURE D1 Performance comparison for recovering differentially abundant features across different scenarios, $p = 11$, $K = 0$. (a) Matthews' correlation coefficient vs. ground truth effect size. (b) False discovery rate vs. ground truth effect size. The dashed line shows the nominal FDR for all methods. (c) True positive rate (power) vs. ground truth effect size.

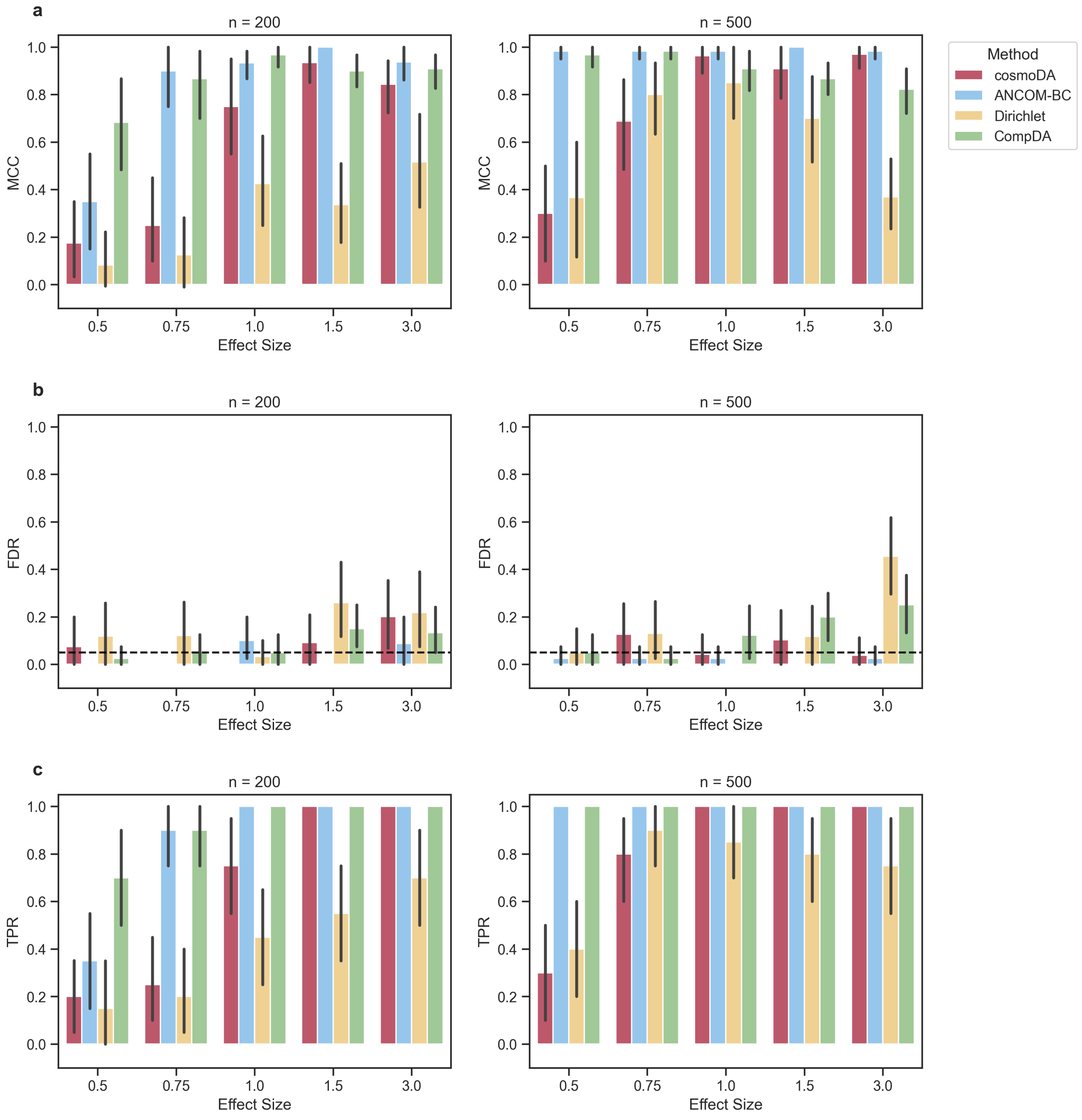

FIGURE E1 Performance comparison for recovering a differentially abundant rare feature across different scenarios, misspecified data generation. (a) Matthews' correlation coefficient vs. ground truth effect size. (b) False discovery rate vs. ground truth effect size. The dashed line shows the nominal FDR for all methods. (c) True positive rate (power) vs. ground truth effect size.

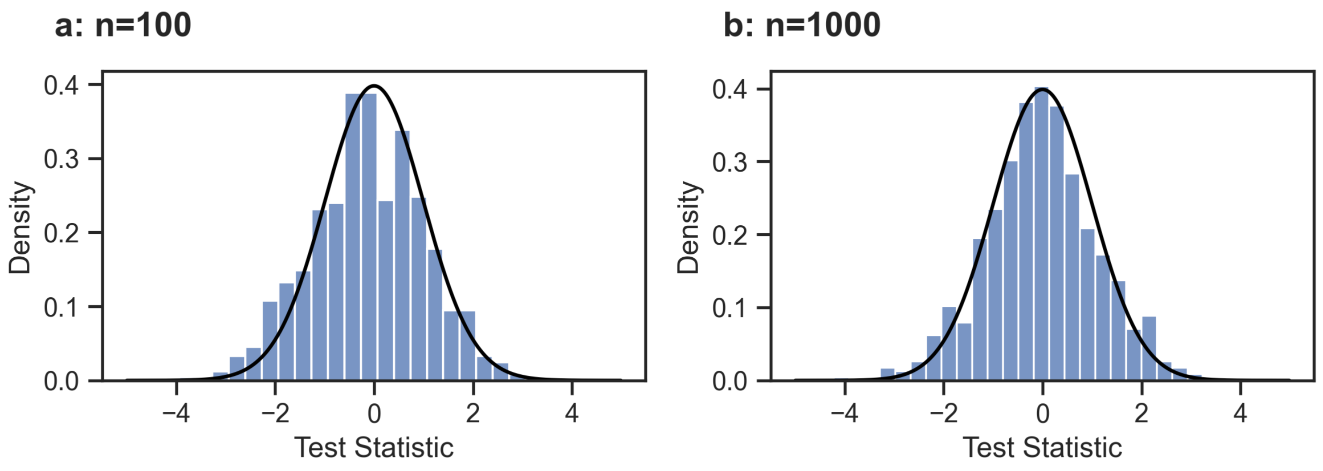

FIGURE F1 Distribution of the test statistic T for the model comparison benchmark with $p = 11$. The blue histogram shows the empirical distribution of test statistics for all non-differentially abundant features over all datasets with (a) $n = 100$ and (b) $n = 1000$. The black curve shows the corresponding density of a t-distribution with $n - 3$ degrees of freedom.

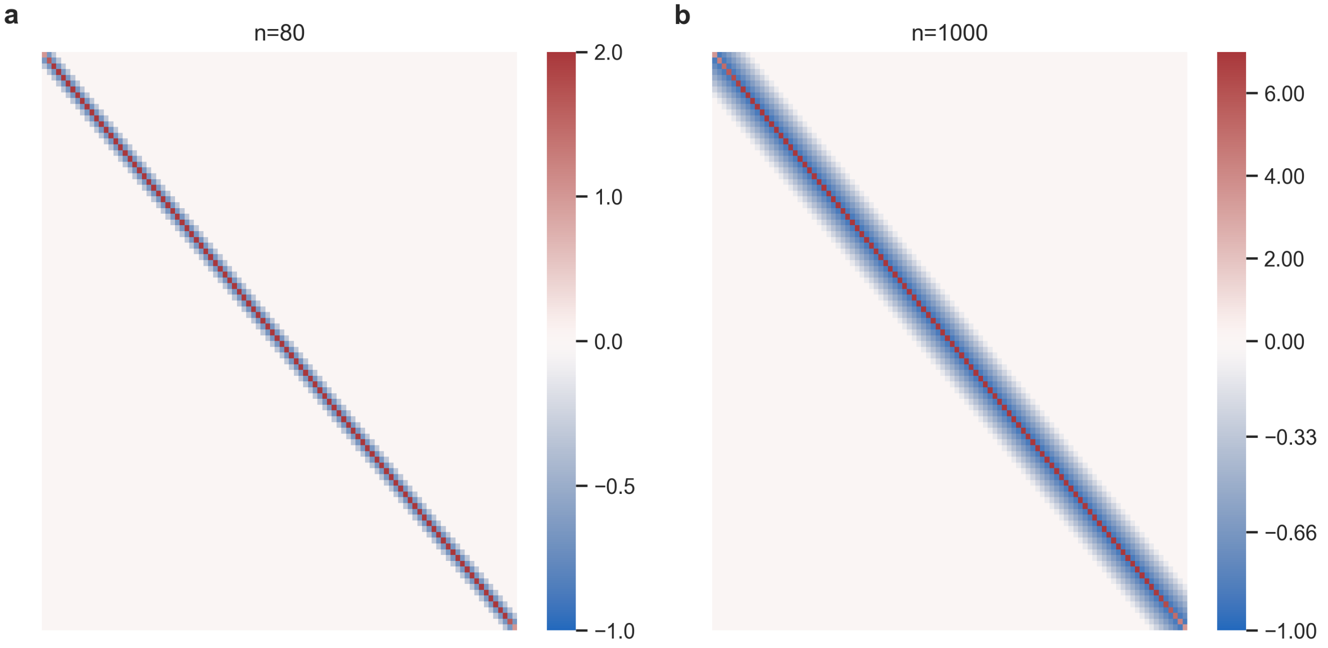

FIGURE F2 Interaction matrices used for data generation in the benchmark testing recovery of K (Section 3.1). (a) $n = 80$, (b) $n = 1000$.

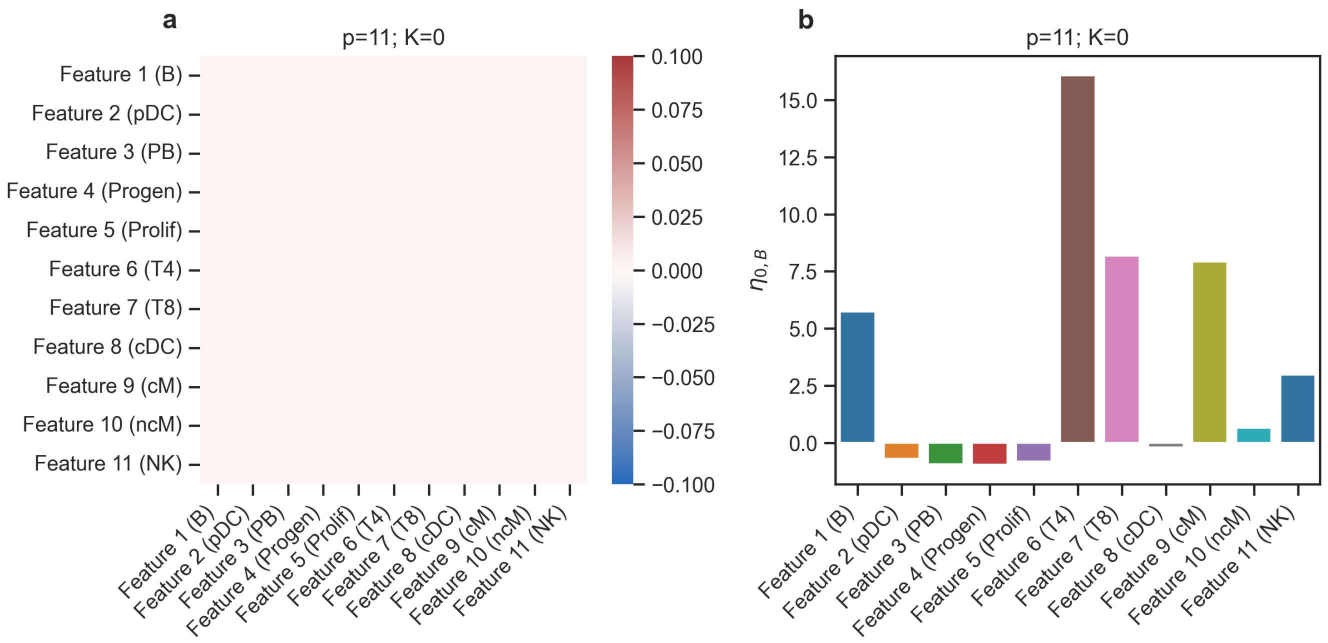

FIGURE F3 Data generation parameters used for the differential abundance testing benchmark (Section 3.2), $K = 0$. (a) Interaction matrix ($\boldsymbol{K}_B$). (b) Location vector ($\boldsymbol{\eta}_{0,B}$).

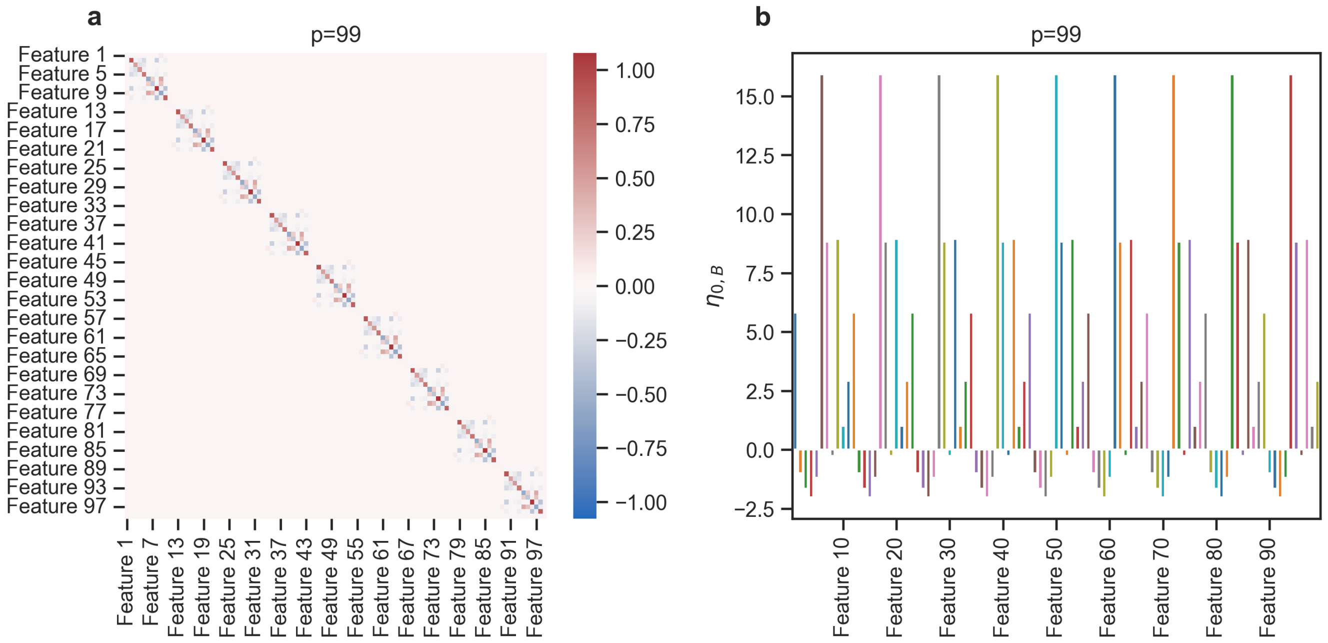

FIGURE F4 Data generation parameters used for the differential abundance testing benchmark (Section 3.2), $p = 99$. (a) Interaction matrix ($\boldsymbol{K}_B$). (b) Location vector ($\boldsymbol{\eta}_{0,B}$).

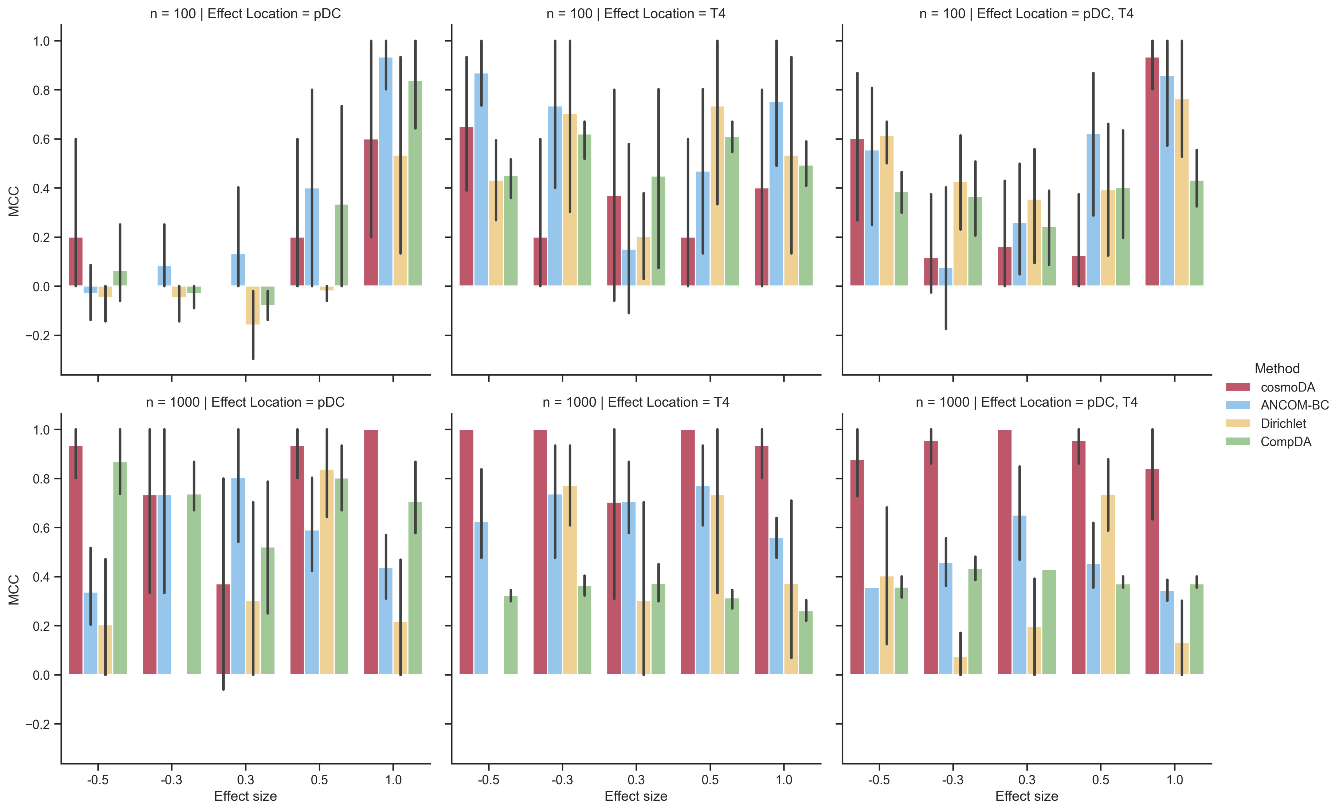

F I G U R E F5 **Detailed breakdown of Matthews' correlation coefficient for the differential abundance testing benchmark (Section 3.2),** $p = 11$**.**

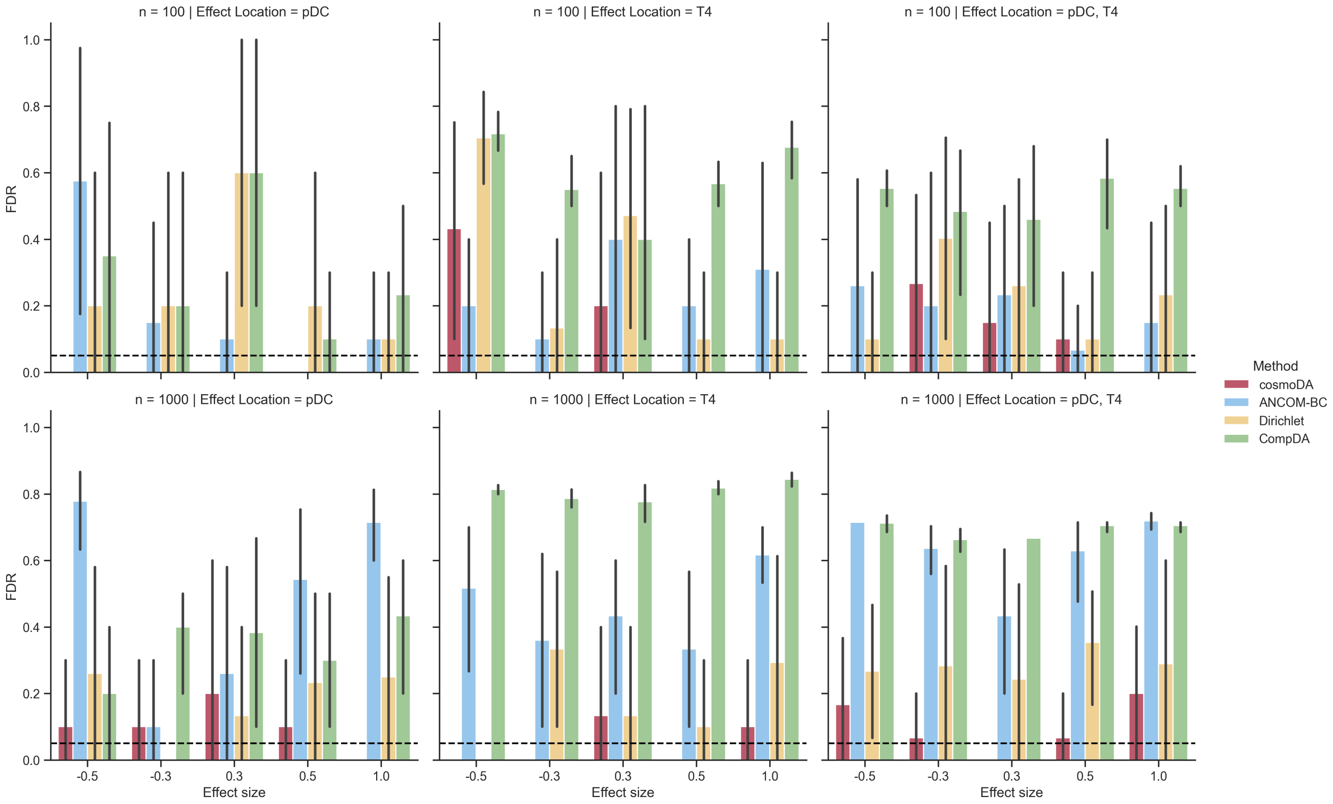

FIGURE F6 Detailed breakdown of false discovery rate for the differential abundance testing benchmark (Section 3.2), $p = 11$. The dashed lines denote the nominal FDR for all methods.

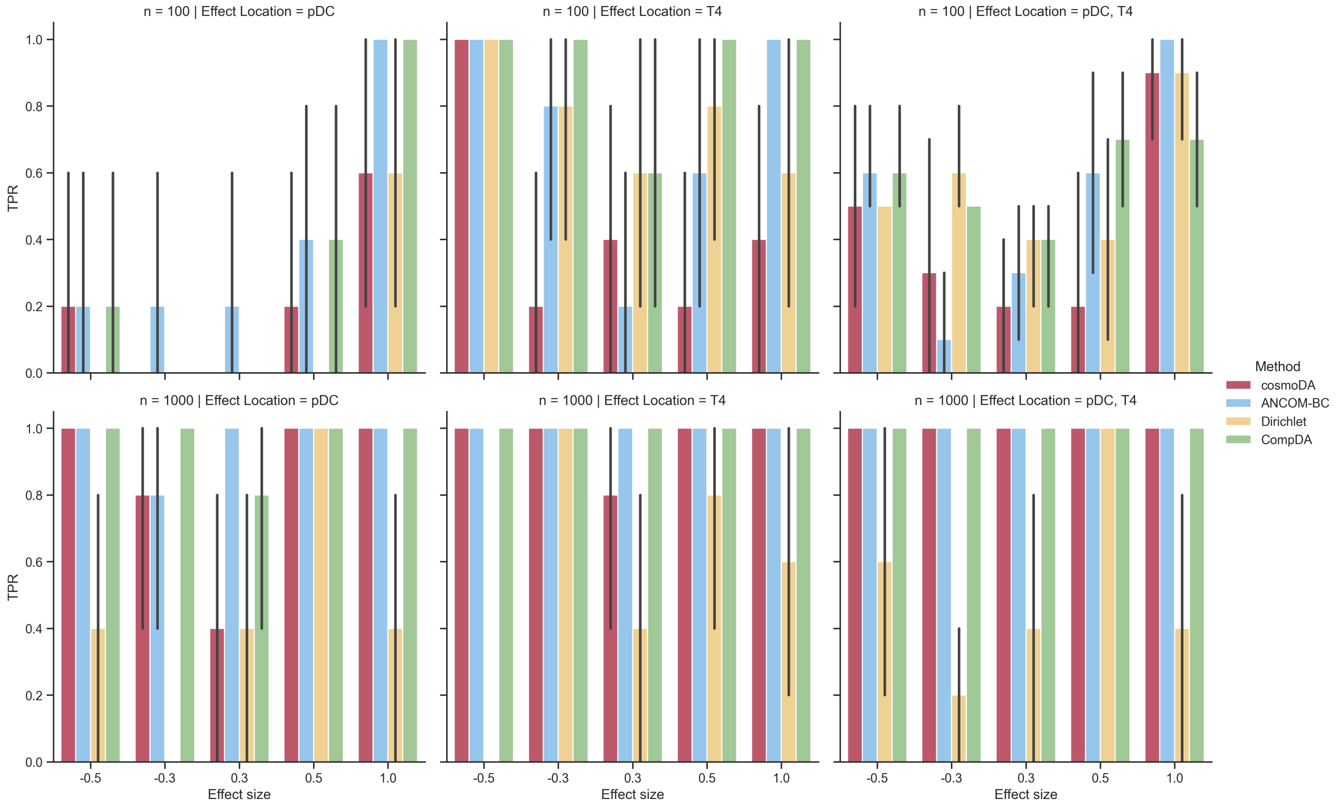

FIGURE F7 Detailed breakdown of power (true positive rate) for the differential abundance testing benchmark (Section 3.2), $p = 11$.

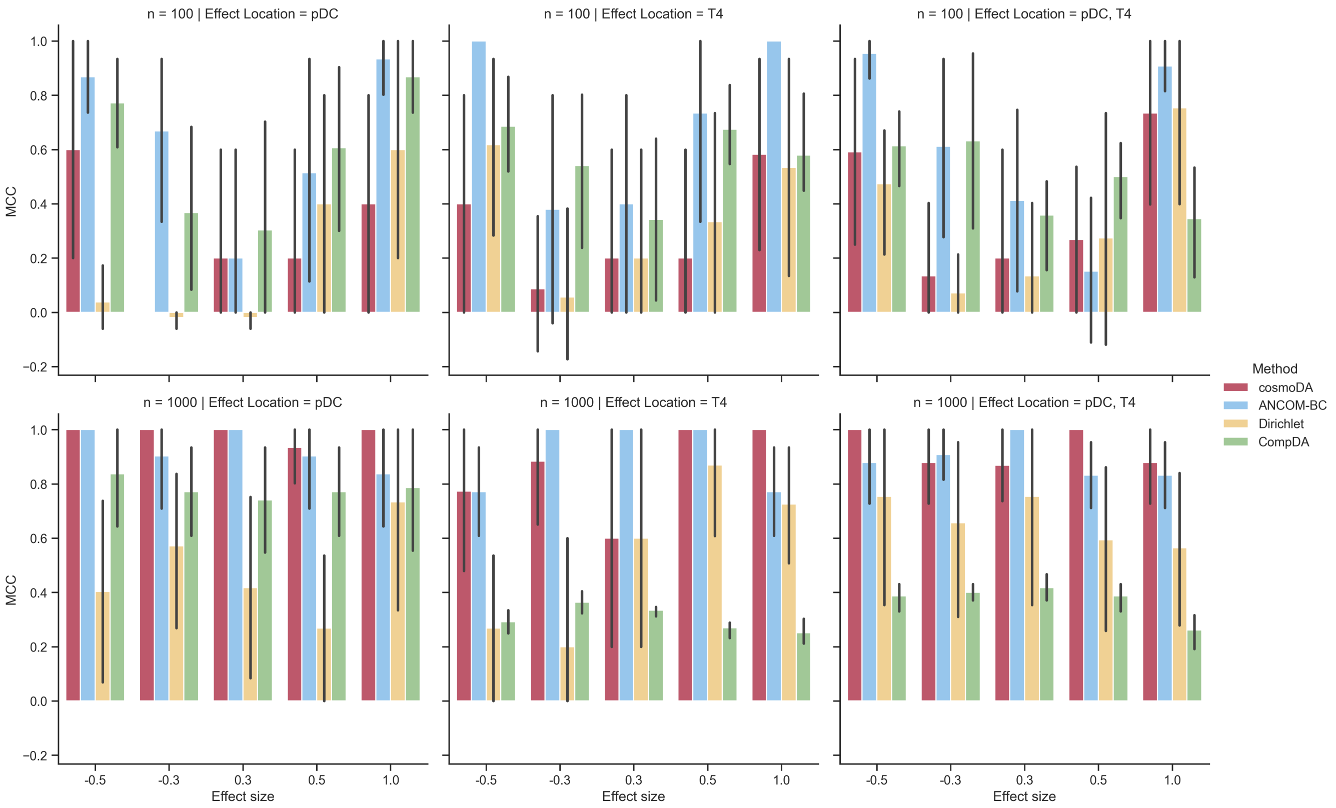

FIGURE F8 Detailed breakdown of Matthews' correlation coefficient for the differential abundance testing benchmark (Section 3.2), $K = 0$.

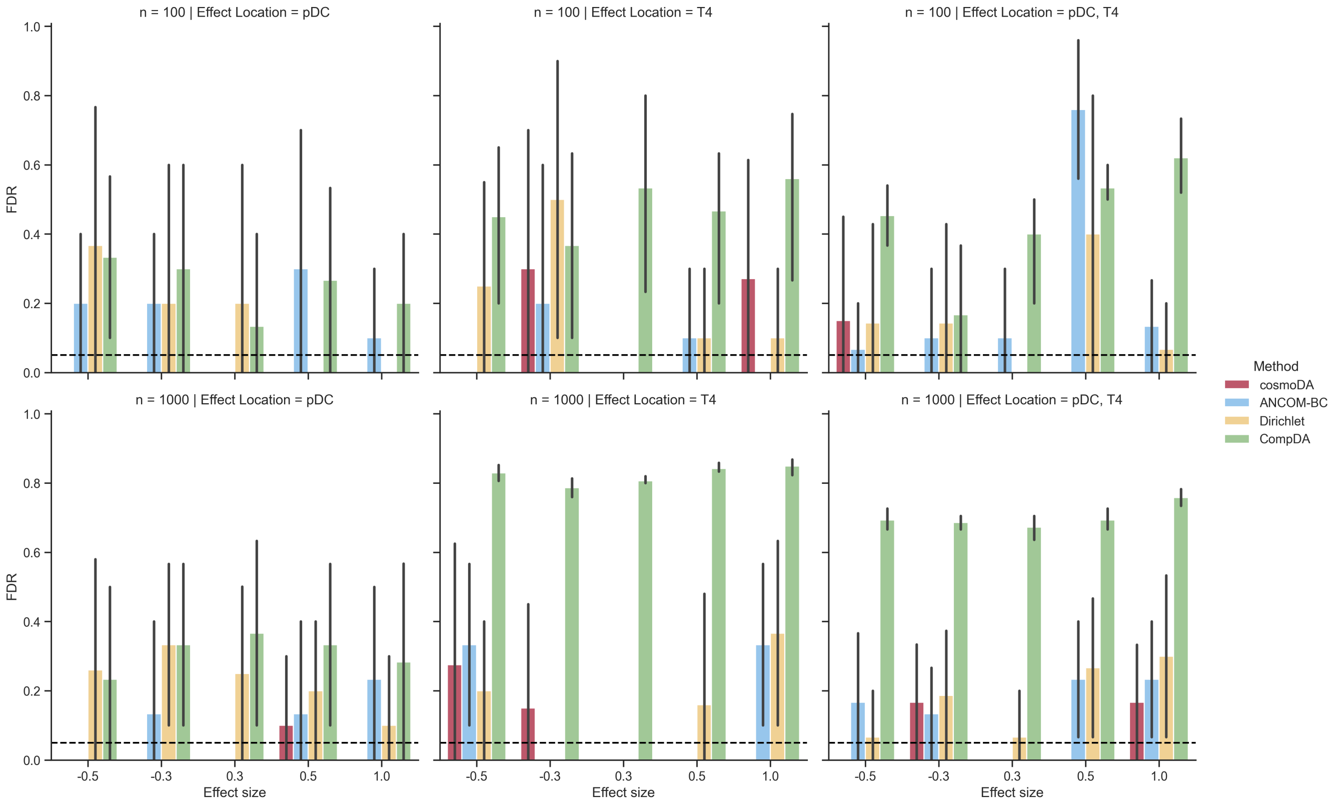

FIGURE F9 Detailed breakdown of false discovery rate for the differential abundance testing benchmark (Section 3.2), $K = 0$. The dashed lines denote the nominal FDR for all methods.

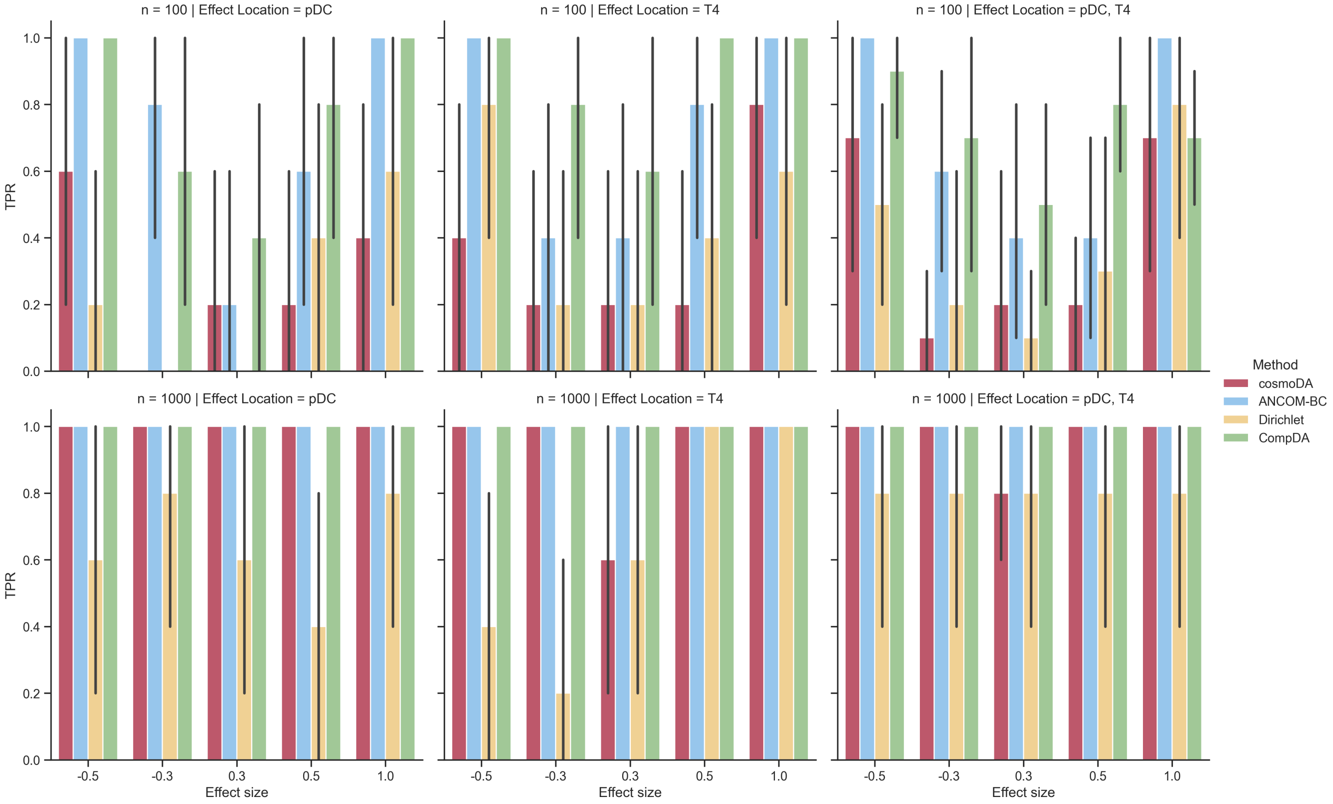

F I G U R E F10 Detailed breakdown of power (true positive rate) for the differential abundance testing benchmark (Section 3.2), $K = 0$.

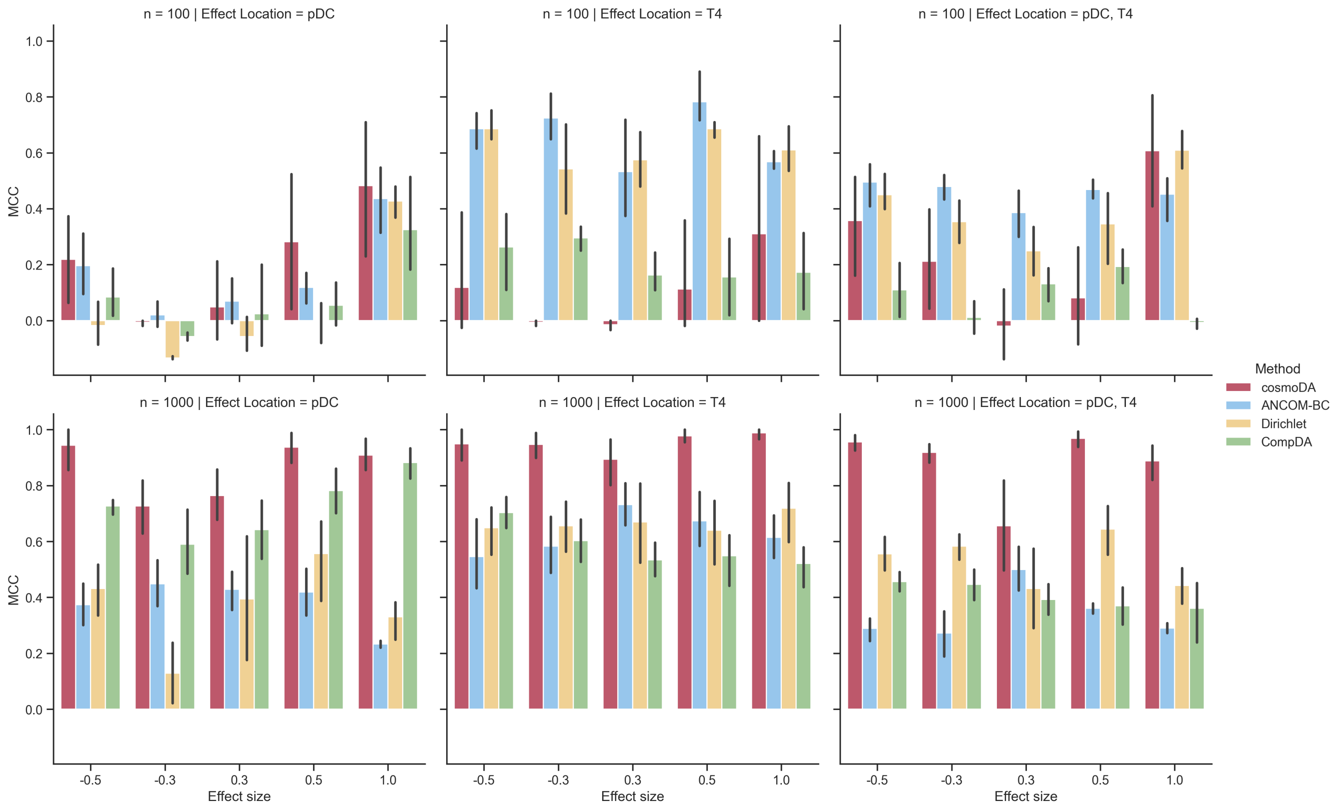

FIGURE F11 Detailed breakdown of Matthews' correlation coefficient for the differential abundance testing benchmark (Section 3.2), $p = 99$.

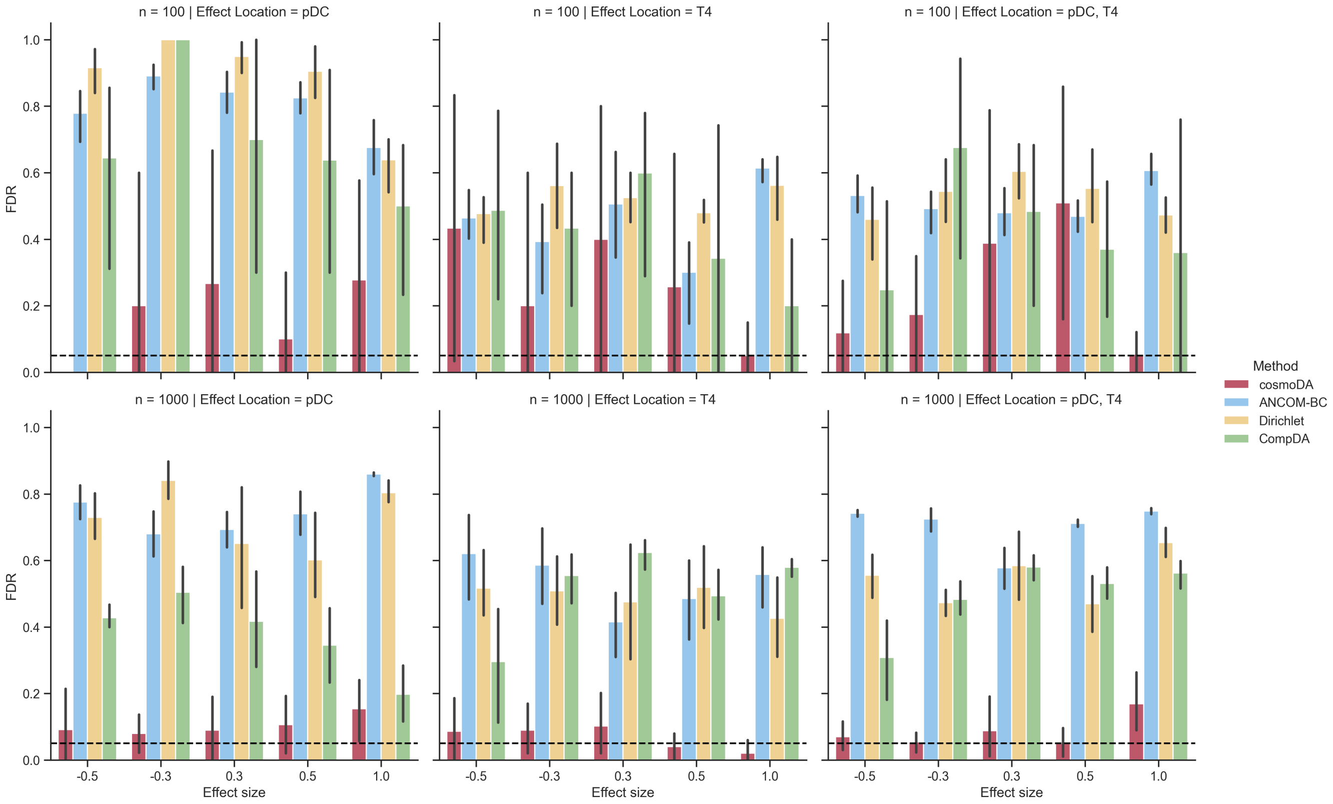

FIGURE F12 Detailed breakdown of false discovery rate for the differential abundance testing benchmark (Section 3.2), p = 99. The dashed lines denote the nominal FDR for all methods.

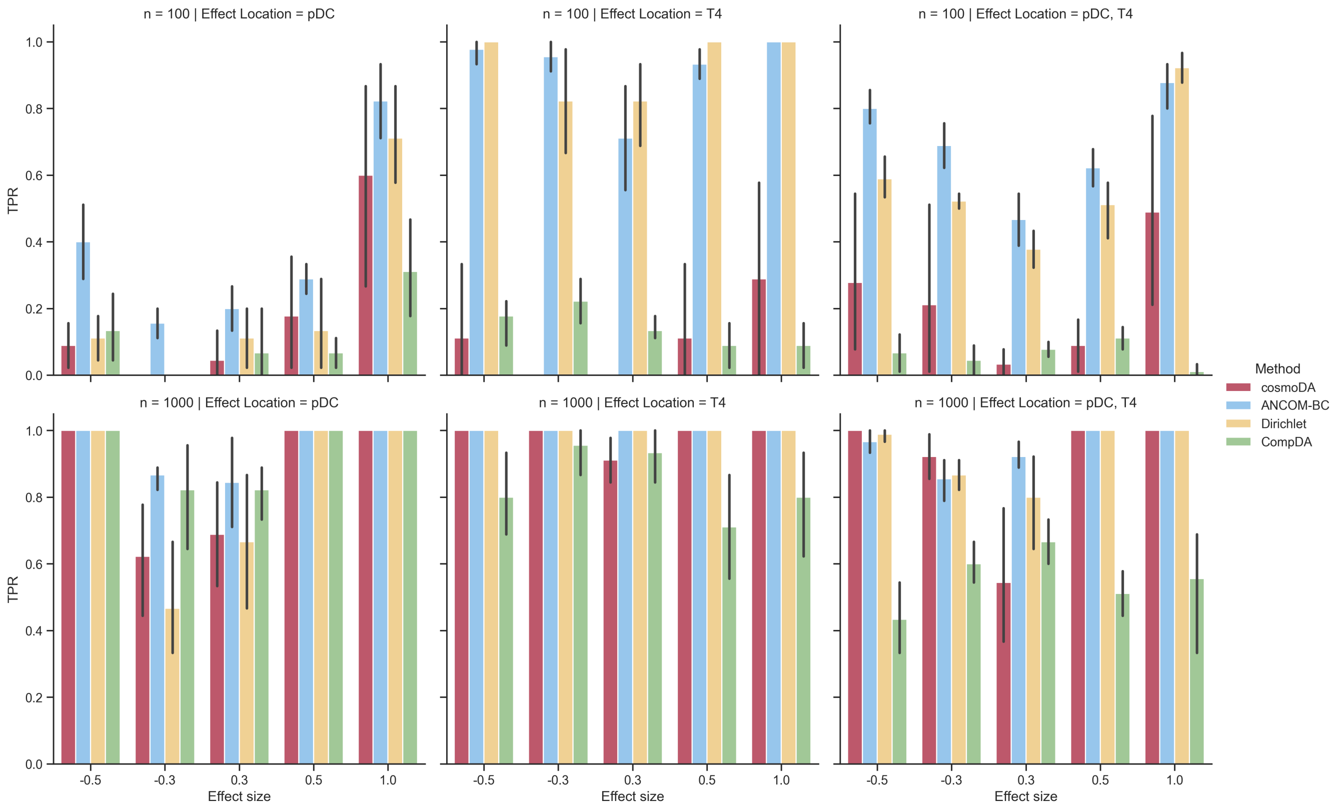

FIGURE F13 Detailed breakdown of power (true positive rate) for the differential abundance testing benchmark (Section 3.2), $p = 99$.

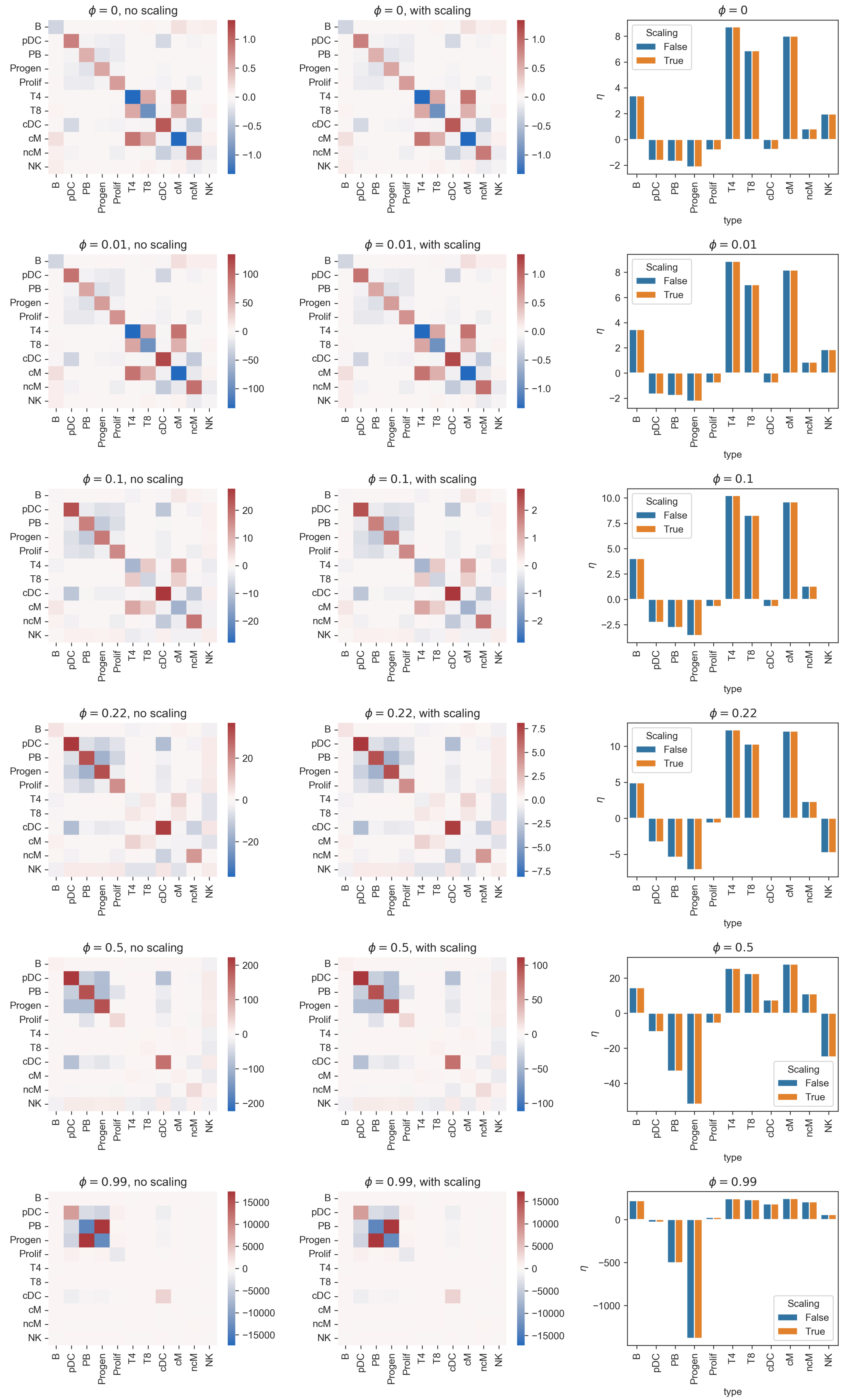

FIGURE F14 Impact of scaling Γ and g on the estimation of K and η. Results shown for the SLA scRNA-seq data[45]. Rows show selected values of the exponent ϕ in the power transformation. Left column: Values of $\boldsymbol{K}$ without scaling. Middle column: Values of $\boldsymbol{K}$ with scaling. Right column: Values of $\boldsymbol{\eta}$ with and without scaling.

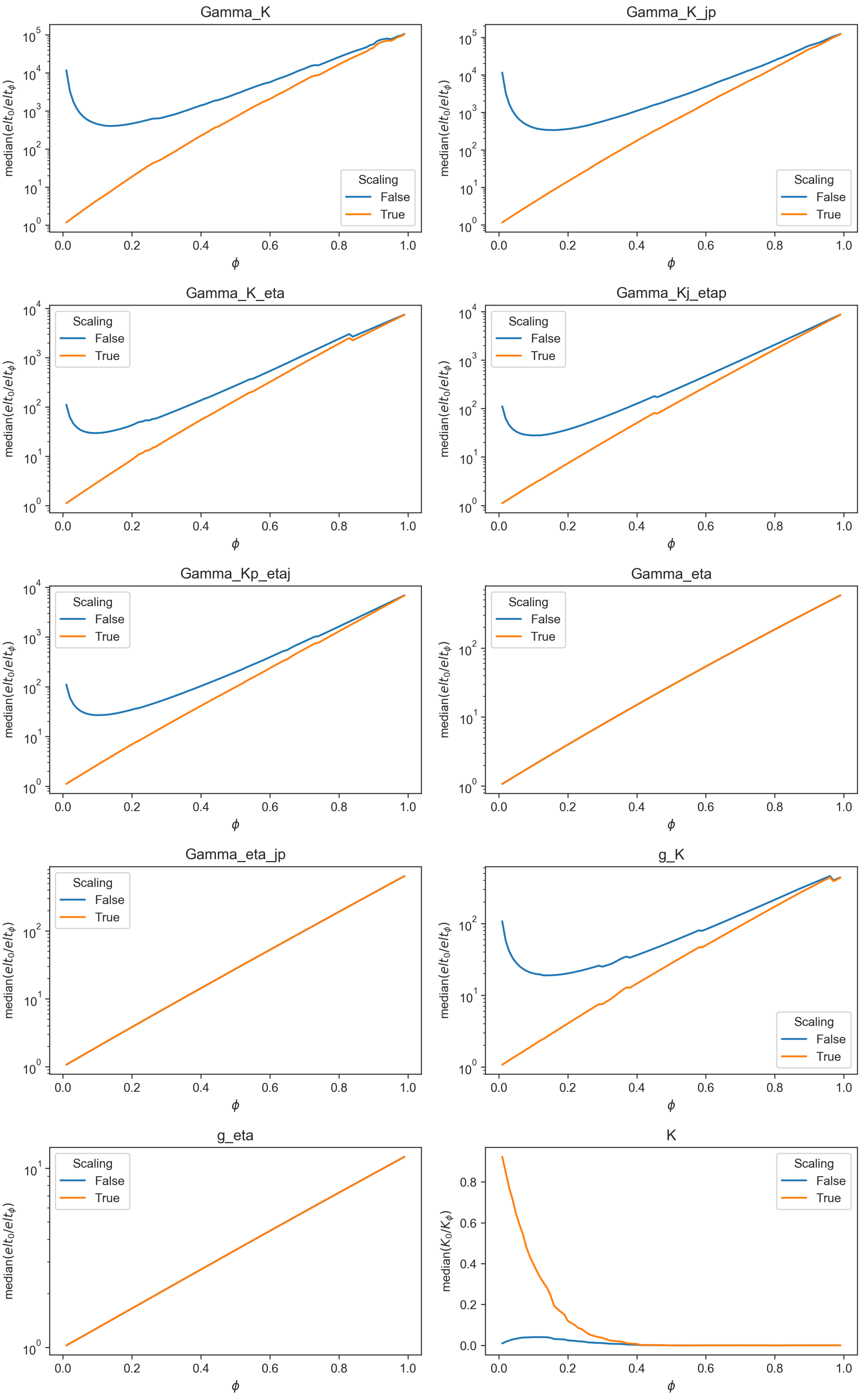

FIGURE F15 Impact of scaling factor for power transforms on the score matching parameters Γ and g and the interaction matrix K. All plots except bottom right show the median entry of $E_{\phi=0}/E_{\phi=\phi'}$ for E being one of the score matching elements in Eq. 11. Bottom right: Same quantity for the estimated interaction matrix $\boldsymbol{K}$.

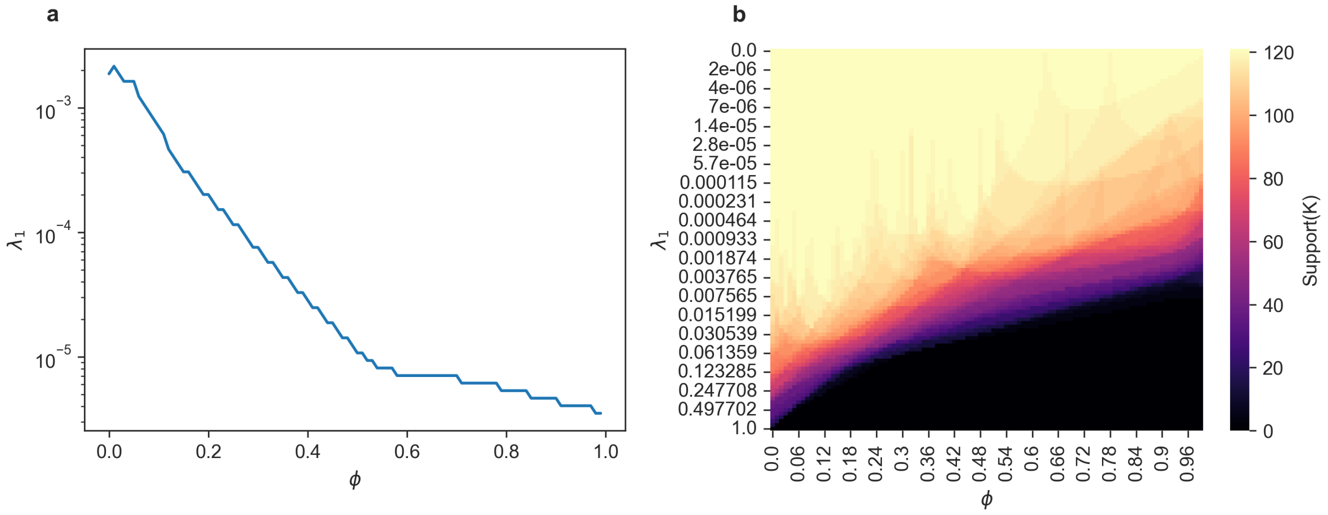

FIGURE F16 Relationship between power and regularization strength for the SLA scRNA-seq data[45]. (a) Value of λ_1 selected through cross validation in relation to exponent ϕ of the power transform. (b) Number of nonzero entries in $\boldsymbol{K}$ for every λ_1 and ϕ.
